# Supplementary material for: Stress-induced OMA1-mediated cleavage of AIFM1 suppresses cell growth by controlling mitochondrial OXPHOS activity
Source: EMBO J. 2026 Mar 24;45(11):3655–98. doi: 10.1038/s44318-026-00734-y (PMC13226697; doi:10.1038/s44318-026-00734-y)
Supplement: Supplementary file 1 — Appendix [file 44318_2026_734_MOESM1_ESM.pdf]

APPENDIX

Stress-induced OMA1-mediated cleavage of AIFM1 suppresses cell growth by controlling mitochondrial OXPHOS activity

Mitsuhiro Nishigori, Serina Hirata, Hidetaka Kosako, Takeshi Ichinohe, Hendrik Nolte, Jan Riemer, Thomas Langer, and Takumi Koshiba✍

✍ koshiba@kyudai.jp

File including

Appendix Table S1 (Page 2) Neo-amino-terminal peptides identified by mass spectrometry.
Appendix Table S2 (Page 3) Summary of exact *p* values (Expanded view figures).
Appendix Figure S1 (Pages 4-6) Biochemical validation of stably expressed OMA1/Myc.
Appendix Figure S2 (Pages 7-8) AIFM1 cleavage site targeted by OMA1 and its non-cleavable variant.
Appendix Figure S3 (Page 9) The role of the AIFM1–MIA40 interaction in OMA1-dependent AIFM1 processing.
Appendix Figure S4 (Pages 10-11) AIFM1 cleavage and cell death.
References (Page 12)

Appendix Table S1 Neo-amino-terminal peptides identified by mass spectrometry.

Gene Symbol	Protein Name	Annotated Peptide Sequence
AIFM1	Apoptosis-inducing factor 1, mitochondrial	[R].VMGLGLSPEE.[K]
AK2	Isoform 2 of Adenylate kinase 2, mitochondrial	[R].GIHCAIDASQTPDIVFASILAAFSKATS.[-]<br>[R].QAEMLDDLME.[K]
ALDH1L2	Mitochondrial 10-formyltetrahydrofolate dehydrogenase	[R].TPQPEEGATYEGIQ.[K]
ALDH4A1	Delta-1-pyrroline-5-carboxylate dehydrogenase, mitochondrial	[K].STGSVVGQQPFGGA.[R]
BCS1L	Mitochondrial chaperone BCS1	[L].SVAPQQSLVLLEDVDAAFLS.[R]
CRLS1	Cardiolipin synthase (CMP-forming)	[K].AAPEPAAGGGGAAAQAPSA.[R]
DNAJA3	DnaJ homolog subfamily A member 3, mitochondrial	[K].QYDAYGSAGFDPGTSSSGQGYW.[R]
GLUD1	Glutamate dehydrogenase 1, mitochondrial	[R].DDGSWEVIEGY.[R]
GOT2	Aspartate aminotransferase, mitochondrial	[A].SSWWTHVEMGPPDPILGVTEAFKRDTNS.[K]
GPT2	Alanine aminotransferase 2	[K].LLEETGICVVPGSGFGQ.[R]
HSPD1	60 kDa heat shock protein, mitochondrial	[I].AEDVDGEALSTLVLN.[R]<br>[K].CEFQDAYVLLSE.[K]
LAP3	Cytosol aminopeptidase	[L].MESPANEMTPT.[R]
MRPL46	39S ribosomal protein L46, mitochondrial	[K].ALTPLQEEMAGLLQQIEVE.[R]<br>[W].MLPQVEWQPGETL.[R]
MRPS7	28S ribosomal protein S7, mitochondrial	[K].AAAATETSSVFADPVIS.[K]
NNT	NAD(P) transhydrogenase, mitochondrial	[R].EANSIVITPGYGLCAA.[K]
PCK2	Phosphoenolpyruvate carboxykinase (GTP), mitochondrial	[R].QCPIMDPAWEAPEGVPIDAIIFGG.[R]
PRDX5	Peroxiredoxin-5, mitochondrial	[K].ATDLLLDDSLVSLFGN.[R]
SFXN3	Sideroflexin-3	[R].AGVATPGLTEDQLW.[R]
STOML2	Stomatin-like protein 2, mitochondrial	[K].ESMQMQVEAE.[R]
SUCLG1	Succinate--CoA ligase (ADP/GDP-forming) subunit alpha, mitochondrial	[F].AAAAINEAIDAEIPLVVCITEGIPQQDMV.[R]<br>[Q].SAGVVVSMSPAQLGTTIY.[K]
TIMM50	Mitochondrial import inner membrane translocase subunit TIM50	[M].IIEPTSPCLLPDPL.[R]
TOMM40	Mitochondrial import receptor subunit TOM40 homolog	[A].SSPPAGPPPPPTPSLVGLPPPPPSPPGFTLPPLGGGLGTGSSTG.[R]<br>[S].SPPAGPPPPPTPSLVGLPPPPPSPPGFTLPPLGGGLGTGSSTG.[R]
UQCRC1	Cytochrome b-c1 complex subunit 1, mitochondrial	[L].QSVPETQVSILDNGL.[R]

Appendix Table S2 Summary of exact *p* values (Expanded view figures).

Figure EV4D

Basal respiration	*P*
WT vs AIFM1^{TCS}	0.2058
AIFM1^{KO} vs AIFM1^{TCS}	< 0.0001
AIFM1^{KO} vs AIFM1$^{TCS/TEV}$	0.0537
AIFM1^{TCS} vs AIFM1$^{TCS/TEV}$	< 0.0001

Maximum respiration	*P*
WT vs AIFM1^{TCS}	0.8393
AIFM1^{KO} vs AIFM1^{TCS}	< 0.0001
AIFM1^{KO} vs AIFM1$^{TCS/TEV}$	0.299
AIFM1^{TCS} vs AIFM1$^{TCS/TEV}$	< 0.0001

ATP production	*P*
WT vs AIFM1^{TCS}	0.9839
AIFM1^{KO} vs AIFM1^{TCS}	< 0.0001
AIFM1^{KO} vs AIFM1$^{TCS/TEV}$	0.7099
AIFM1^{TCS} vs AIFM1$^{TCS/TEV}$	< 0.0001

Figure EV4E

mtDNA : nDNA	*P*
WT vs AIFM1^{KO}	0.9717
AIFM1^{KO} vs AIFM1^{TCS}	0.9698
AIFM1^{TCS} vs AIFM1$^{TCS/TEV}$	0.9828

Figure EV6E

U.I. vs PR8	*P*
HK2	< 0.0001
GPI	0.0266
PFKP	< 0.0001
ALDOA	0.5868
TPI1	0.0004
GAPDH	0.2127
PGK1	0.0017
PGAM1	< 0.0001
ENO1	< 0.0001
PKM	0.0003

Figure EV4G

two-way ANOVA	*P*
DMSO vs FCCP	< 0.0001

Tukey's test	*P*
DMSO (WT vs AIFM1^{KO})	0.968
DMSO (AIFM1^{KO} vs AIFM1^{TCS})	0.958
DMSO (AIFM1^{TCS} vs AIFM1$^{TCS/TEV}$)	0.782

Figure EV5D

siCtrl vs siTIM23	*P*
FCCP	0.0003

Figure EV6C

GO term	*P*-value ($-\log_{10}$)
Innate immune response	58.1778
Defense response to virus	31.3872
Response to bacterium	24.8962
Inflammatory response	20.6421
Cellular response to IFN-β	18.4353
Defense response to bacterium	16.0168
Antiviral innate immune response	14.0851
Response to virus	13.7878
Activation of innate immune response	12.4401
Cellular response to LPS	12.1481

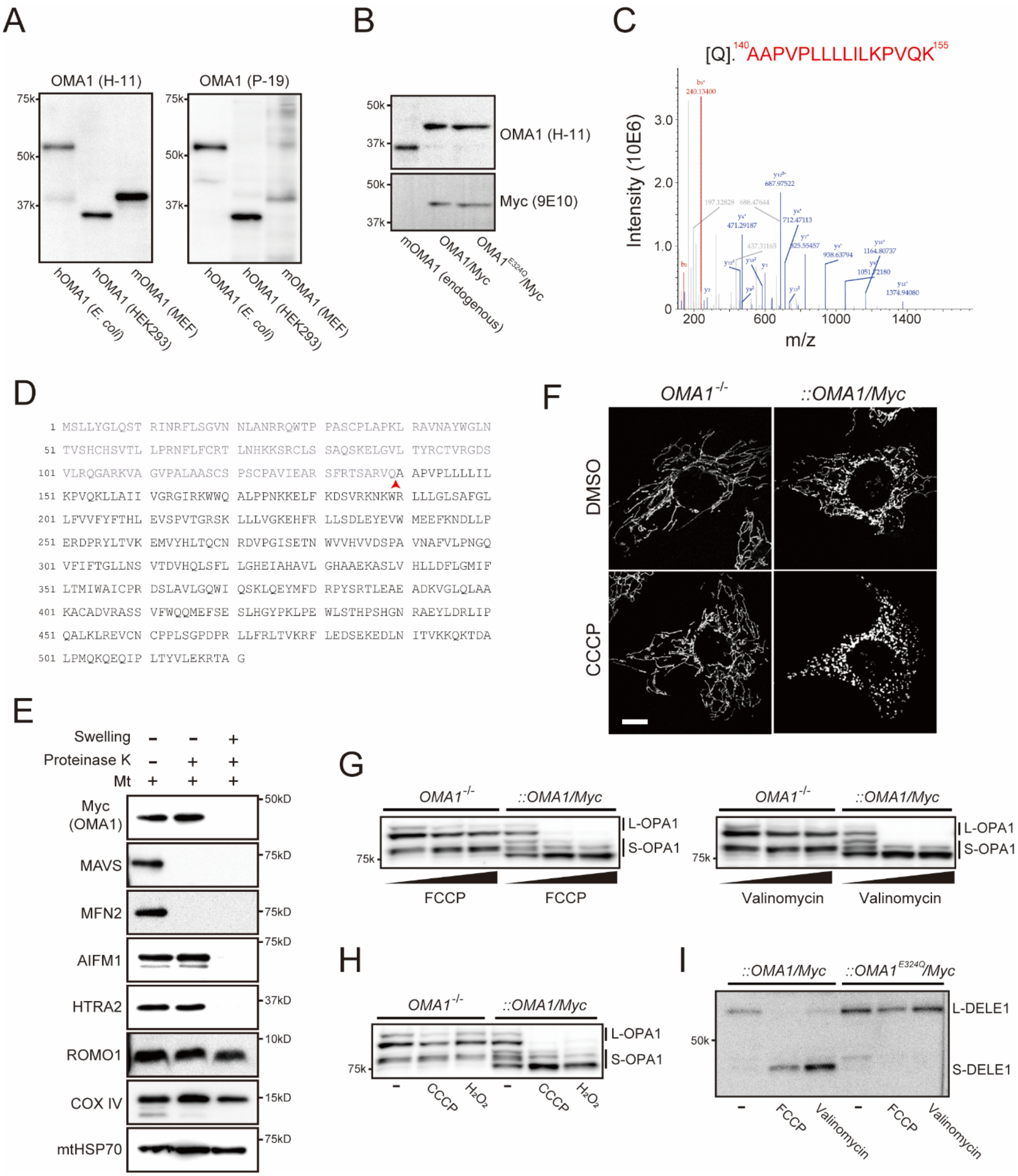

Appendix Figure S1 Biochemical validation of stably expressed OMA1/Myc.

A Constitutive maturation of OMA1 in mitochondria. The molecular size of OMA1 expressed bacterially (hOMA1) or endogenously in HEK293 (hOMA1) or MEF (mOMA1) cells was compared by SDS-PAGE and Western blotting analysis using anti-OMA1 antibodies (left blot, H-

11; right blot, P-19). Size differences in OMA1 between recombinant and endogenous OMA1 show that it is constitutively converted into mature forms after import into mitochondria.

B Western blot analysis of the stably expressed Myc-tagged version of WT OMA1 and its E324Q variant in *OMA1*-null MEFs used in the study. These recombinants show constitutive conversion to their mature form as well as to the endogenous (WT MEFs) protein (left lane). The size difference between the endogenous and recombinant proteins indicates the weight of the Myc tag (4-kDa) fused to the C-terminus of each recombinant protein.

C, D The N-terminal sequence of OMA1/Myc was determined by MS. Mitochondrial extract from *OMA1*$^{-/-}$ MEFs stably expressing OMA1/Myc was immunoprecipitated with anti-Myc antibody, followed by tryptic digestion and LC-MS/MS to identify its N-terminal peptide sequence. The MS/MS spectrum suggested that the most distal N-terminal residue was A140 (red peptide sequence in [C], ^{140}AAPVPLLLLILKPVQK^{155}) identical to that of endogenous OMA1 (Baker *et al*, 2014). Mature OMA1 is generated upon proteolytic cleavage at the site indicated by the red arrowhead in (D).

E Submitochondrial localization of OMA1/Myc demonstrating its proper localization in the IMS. Mitochondria isolated from *OMA1*$^{-/-}$ MEFs stably expressing OMA1/Myc were treated with proteinase K (50 µg/mL) under either isotonic (−SW) or hypotonic swelling (+SW) buffer conditions and kept on ice for 15 min. The reactants were developed by immunoblotting with antibodies against Myc or various mitochondrial membrane markers as indicated. MOM proteins: MAVS and MFN2. IMS proteins: AIFM1 and HTRA2. MIM proteins: ROMO1 and COX IV. Matrix protein: mtHSP70.

F Mitochondrial morphologies under normal or depolarized conditions. *OMA1*$^{-/-}$ or *OMA1*$^{-/-}$ MEFs stably expressing OMA1/Myc were treated with either DMSO or CCCP, and their mitochondrial morphologies were visualized by immunofluorescence microscopy using mtHSP70 antibody. OMA1/Myc controls mitochondrial dynamics upon mitochondrial stress induced by CCCP. Scale bar, 10 µm.

G, **H** *OMA1*$^{-/-}$ or *OMA1*$^{-/-}$ MEFs stably expressing OMA1/Myc were treated for 3 h with FCCP (G, left blot, 0, 20, and 40 µM), valinomycin (G, right blot, 0, 0.1, and 1 µg/mL), or H_2O_2 (H, 1 mM) and analyzed by immunoblotting with OPA1 antibody. The proteolytic activity of OMA1/Myc was indistinguishable from that of the endogenous protein and could be used to monitor OMA1-mediated constitutive OPA1 processing under steady state conditions and stress-inducible OMA1 activations. L- and S-OPA1 indicate long- and short-form OPA1, respectively.

I *OMA1*$^{-/-}$ MEFs stably co-expressing both Myc-tagged versions of OMA1 variants (WT and E324Q) and HA-tagged DELE1 were treated for 3 h with either FCCP (40 µM) or valinomycin (1 µg/mL) and analyzed by immunoblotting with an HA antibody to monitor DELE1-processing in the cells. OMA1/Myc, but not the proteolytically inactive OMA1^{E324Q}/Myc, rescued cells

processed DELE1 when mitochondrial stress was induced. L- and S- DELE1 indicate long and short form DELE1, respectively.

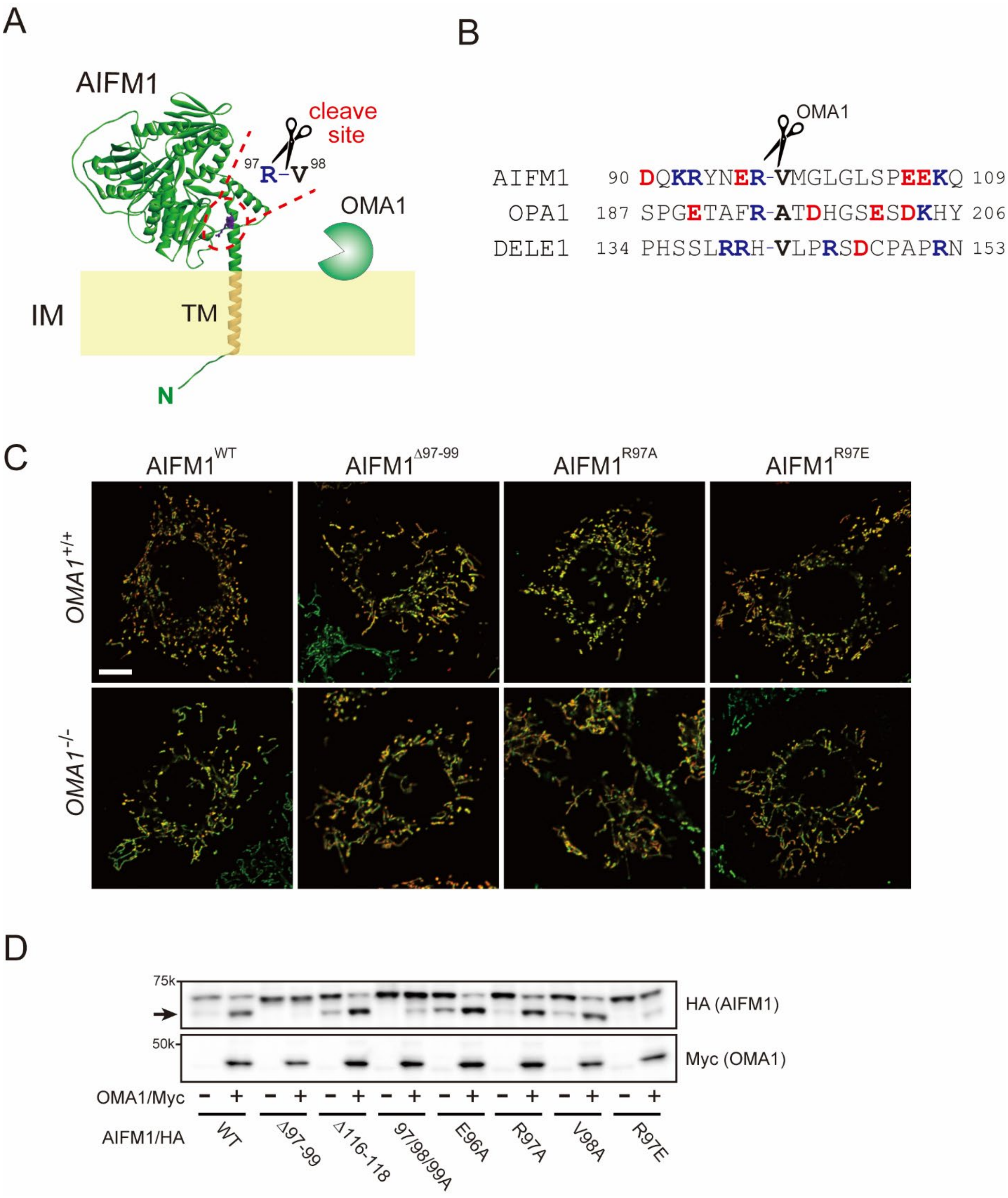

Appendix Figure S2 AIFM1 cleavage site targeted by OMA1 and its non-cleavable variant.
A Topologic model of the cleavage site of AIFM1 targeted by OMA1. The cleavage site (between residues Arg97 and Val98) is located just downstream of the transmembrane (TM) domain in MIM-anchored AIFM1 and is depicted as a stick model (purple) in the structure. The structure of the

mature form of murine AIFM1 (amino acids 55 to 612) as assigned by the AlphaFold3 program (Jumper *et al*, 2021).

B Sequence alignment of OMA1 substrates in *Mus musculus*. No conservation of any OMA1 cleavage motif is observed among these substrates.

C The indicated Myc-labeled WT or AIFM1 variants (Δ97-99, R97A, and R97E) were stably expressed in either *OMA1*$^{+/+}$ or *OMA1*$^{-/-}$ MEFs by a retroviral system. Immunofluorescence against the Myc epitope was used to identify AIFM1 variants in cells and to determine their subcellular localization (red). Mitochondria in the same cells were also identified by staining with anti-mtHSP70 antibody (green). Both AIFM1 and mtHSP70 in mitochondria were completely merged (yellow) in all cells observed. Scale bar, 10 μm.

D HA-tagged plasmids encoding WT AIFM1 or its variants were co-transfected with the Myc-tagged version of OMA1 into HEK293 cells. OMA1-mediated AIFM1 processing was analyzed by Western blotting with anti-HA monoclonal antibody. The arrow indicates the cleaved AIFM1 band.

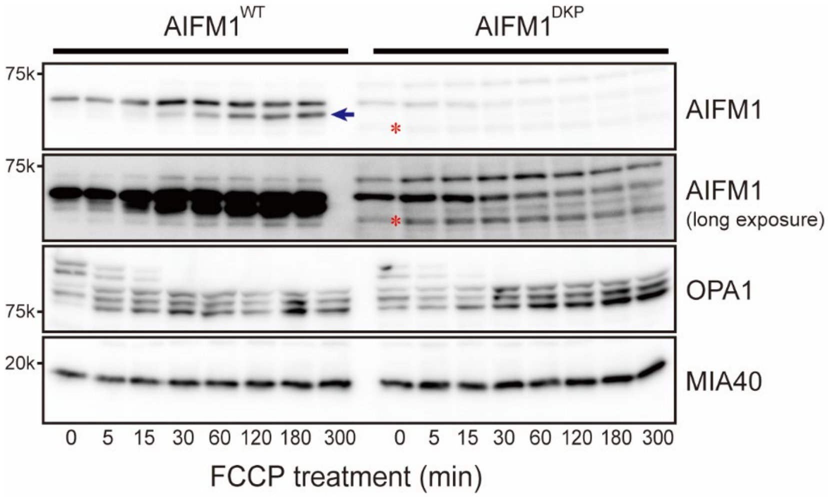

Appendix Figure S3 The role of the AIFM1–MIA40 interaction in OMA1-dependent AIFM1 processing.

AIFM1^{WT}- and AIFM1^{DKP}-expressing cells were incubated in 20 µM FCCP and collected at the indicated time points (0, 5, 15, 30, 60, 120, 180, and 300 min) for Western blot analysis. Note that MIA40 levels do not change between the two cell types. The blue arrow indicates the original AIFM1 band processed by OMA1, and the red asterisk shows the new degradation band in the AIFM1^{DKP} variant.

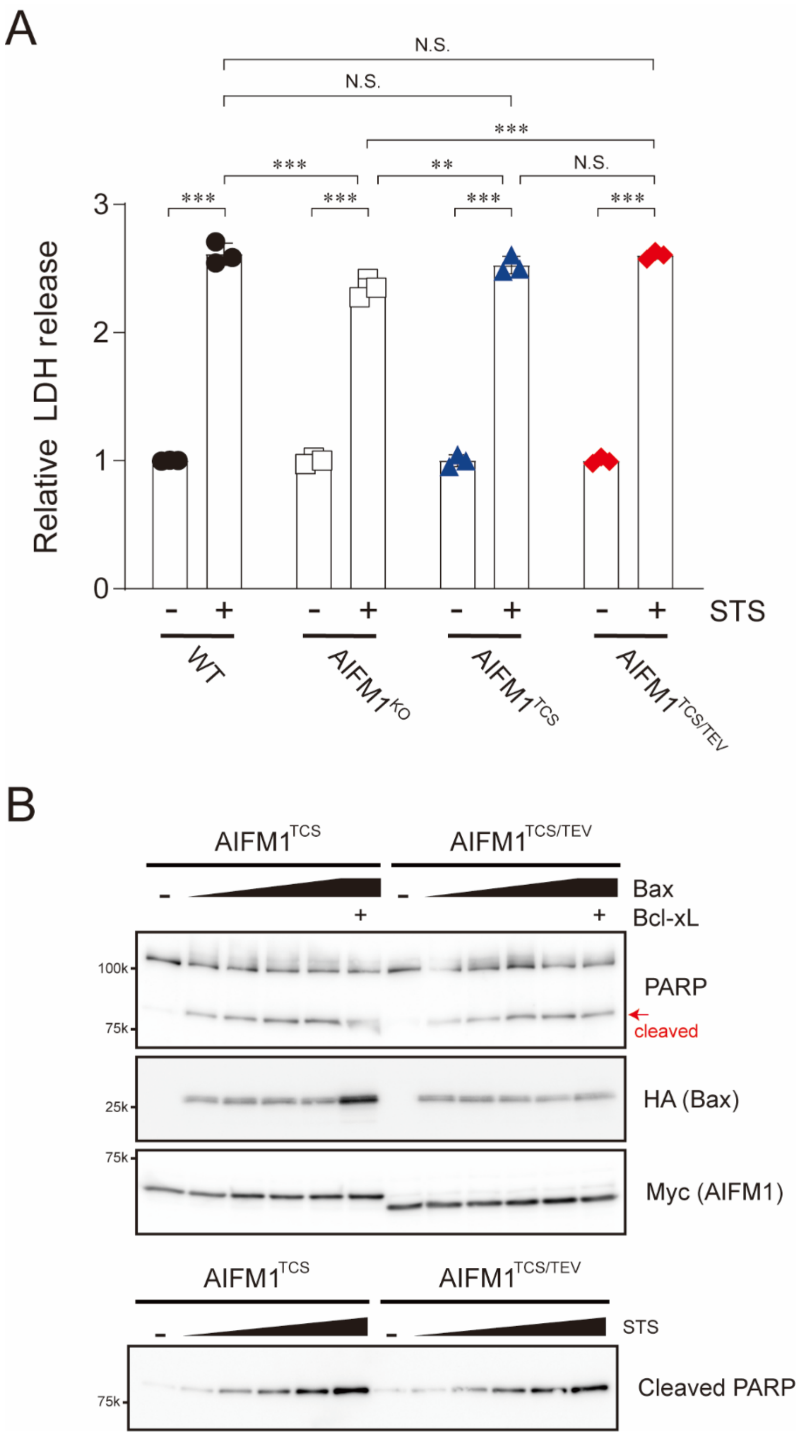

Appendix Figure S4 AIFM1 cleavage and cell death.

A Lactate dehydrogenase (LDH) release from WT, *AIFM1* KO, and AIFM1 variant Flp-In-293 cells treated with DMSO (-) or STS (5 μM) for 24 h. Data are normalized to the relative LDH

release of DMSO-treated cells as 1. Data shown are mean ± SD ($n = 3$). **$p < 0.01$, ***$p < 0.001$, and N.S., not significant.

B Various stimuli of the intrinsic pathway of apoptosis were examined in AIFM1^{TCS}- and AIFM1$^{TCS/TEV}$-expressing cells. The top panels show that cell lines were transfected with increasing amounts of a HA-tagged Bax plasmid (50, 100, 250, or 500 ng) or an empty vector (-) control. The cell lysates were analyzed by SDS-PAGE and immunoblotting 16 h later using antibodies. The right lanes show cells that were co-transfected with a Bcl-xL expression plasmid. The bottom blot shows that AIFM1^{TCS}- or AIFM1$^{TCS/TEV}$-expressing cells were treated with DMSO (-) or an increasing amount of STS (0.1, 1, 2.5, 5, or 10 μM) for 3 h, after which PARP cleavage was detected.

References

Baker MJ, Lampe PA, Stojanovski D, Korwitz A, Anand R, Tatsuta T, Langer T (2014) Stress-induced OMA1 activation and autocatalytic turnover regulate OPA1-dependent mitochondrial dynamics. *EMBO J* 33: 578–593

Jumper J, Evans R, Pritzel A, Green T, Figurnov M, Ronneberger O, Tunyasuvunakool K, Bates R, Žídek A, Potapenko A *et al* (2021) Highly accurate protein structure prediction with AlphaFold. *Nature* 596: 583–589
