# Supplementary figures and images for: Stress-induced OMA1-mediated cleavage of AIFM1 suppresses cell growth by controlling mitochondrial OXPHOS activity
Source: EMBO J. 2026 Mar 24;45(11):3655–98. doi: 10.1038/s44318-026-00734-y (PMC13226697; doi:10.1038/s44318-026-00734-y)

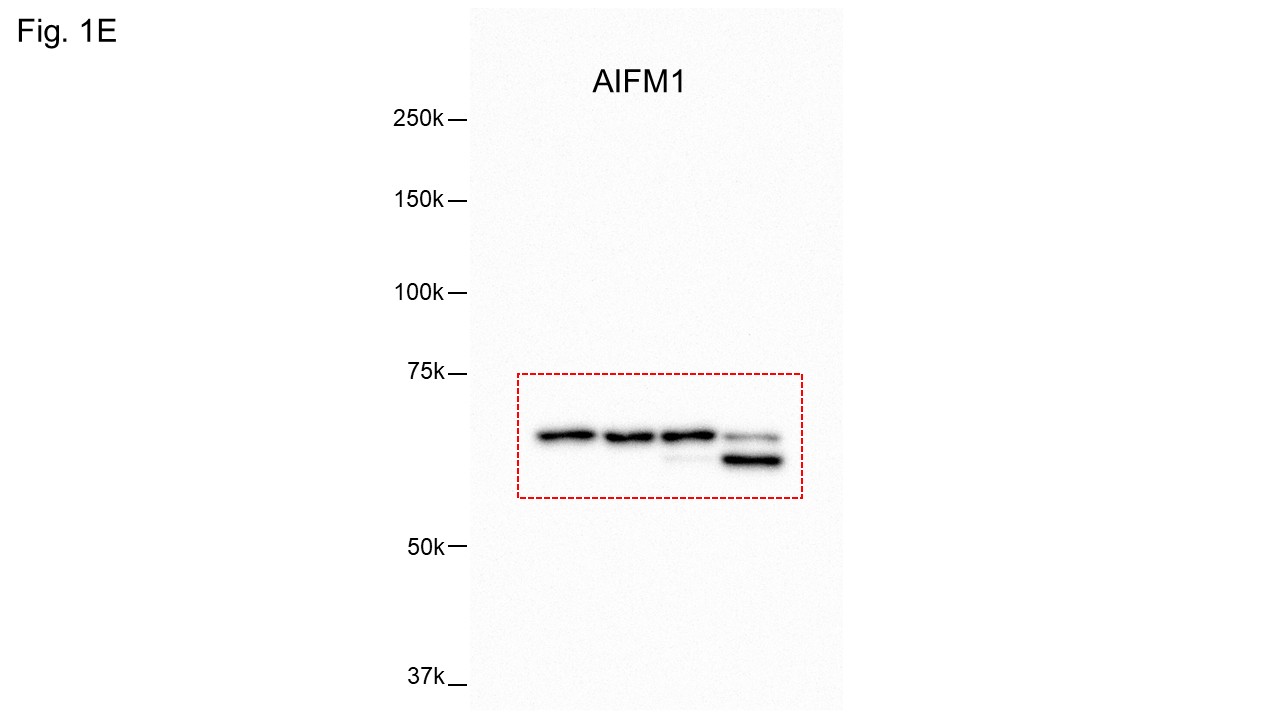

Supplement: Supplementary file 9 — Source data Fig. 1 [file 44318_2026_734_MOESM9_ESM.zip › Source Data Figure 1/1E/1E_blot.TIF]

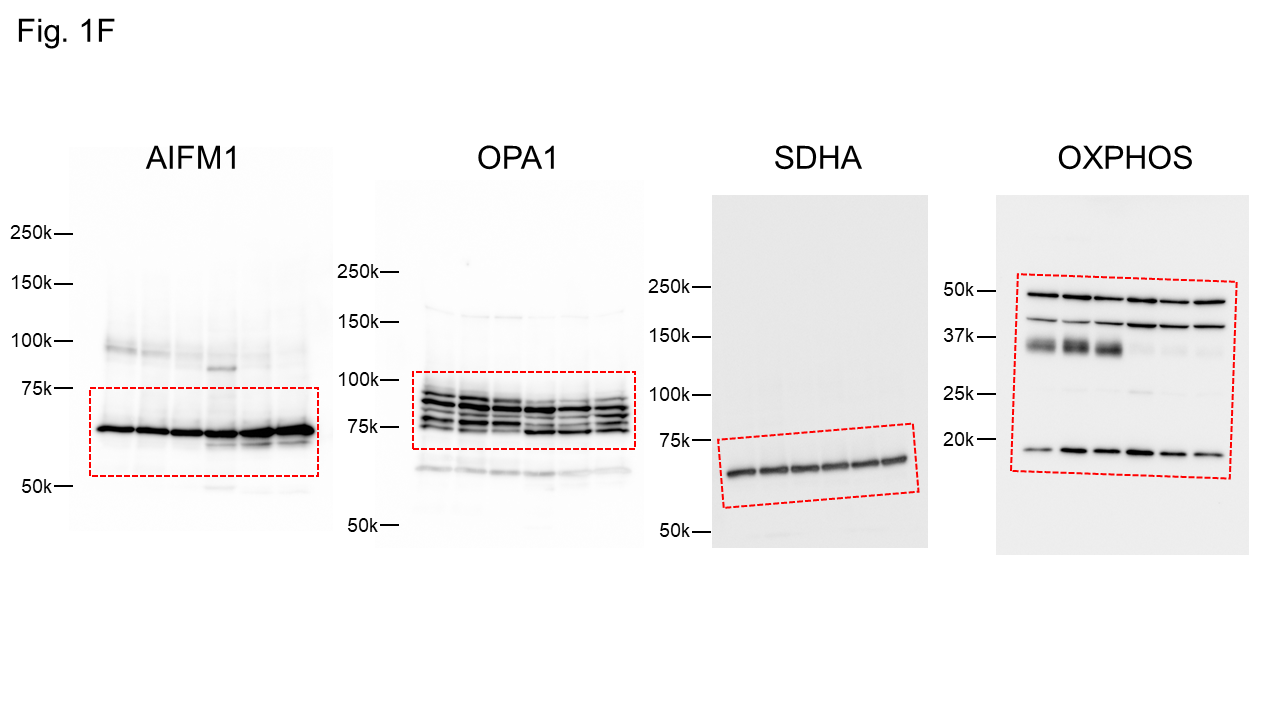

Supplement: Supplementary file 9 — Source data Fig. 1 [file 44318_2026_734_MOESM9_ESM.zip › Source Data Figure 1/1F/1F_blot.TIF]

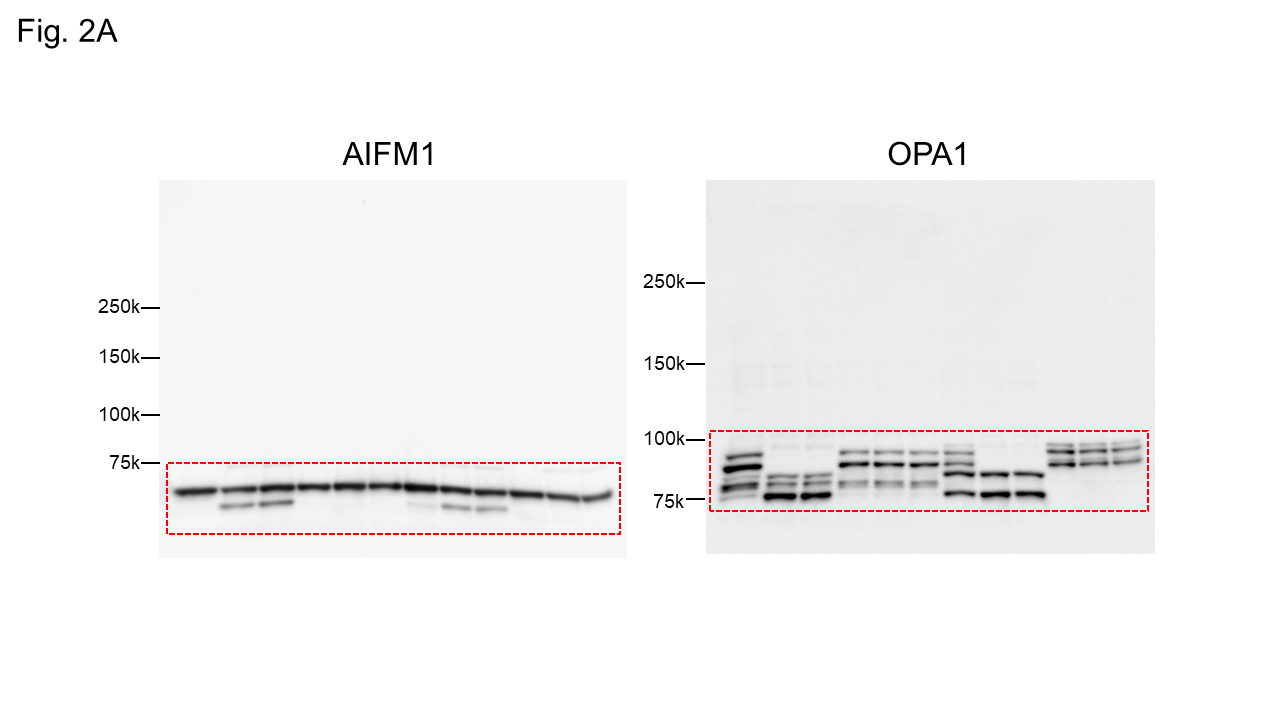

Supplement: Supplementary file 10 — Source data Fig. 2 [file 44318_2026_734_MOESM10_ESM.zip › Source Data Figure 2/2A/2A_blot.TIF]

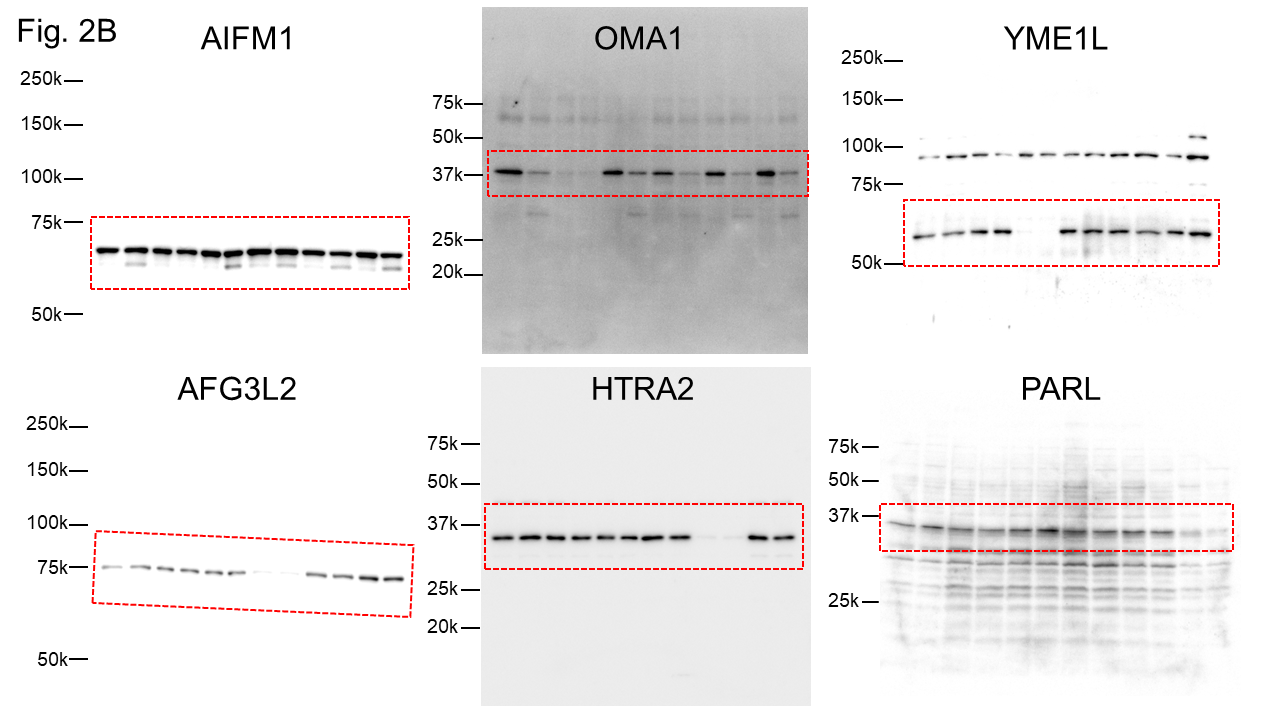

Supplement: Supplementary file 10 — Source data Fig. 2 [file 44318_2026_734_MOESM10_ESM.zip › Source Data Figure 2/2B/2B_blot.TIF]

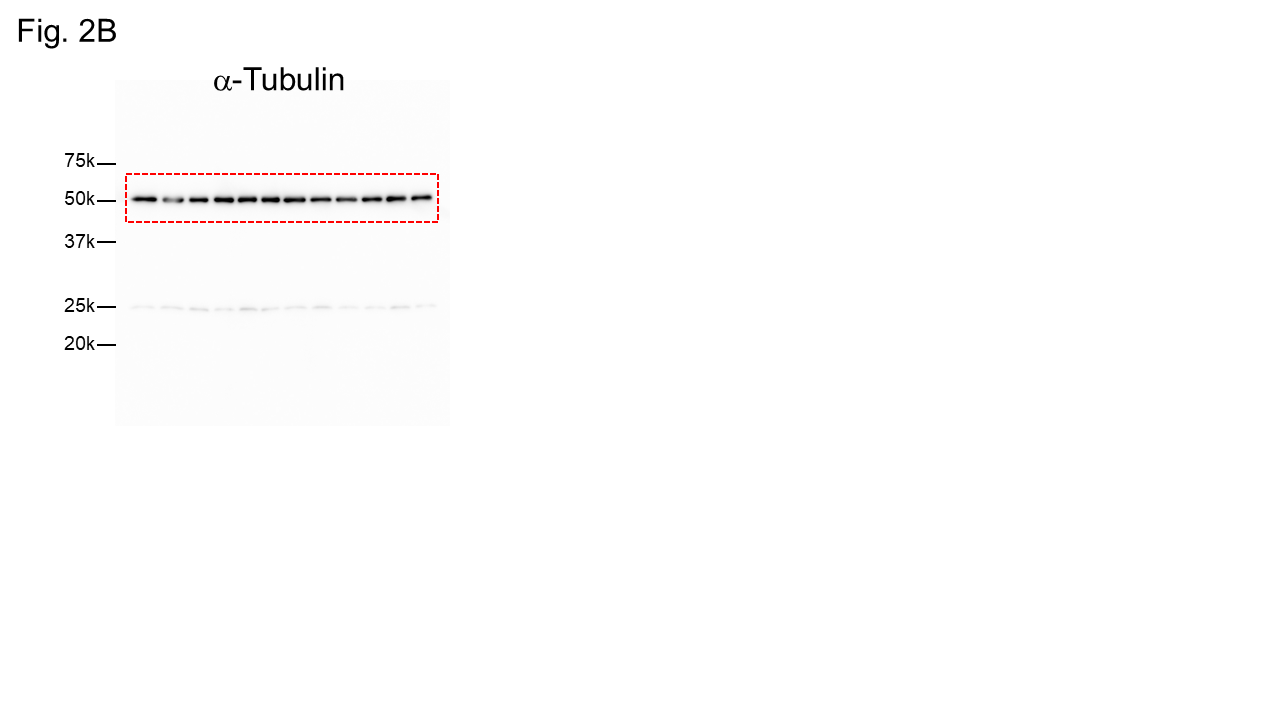

Supplement: Supplementary file 10 — Source data Fig. 2 [file 44318_2026_734_MOESM10_ESM.zip › Source Data Figure 2/2B/2B_tubulin_blot.TIF]

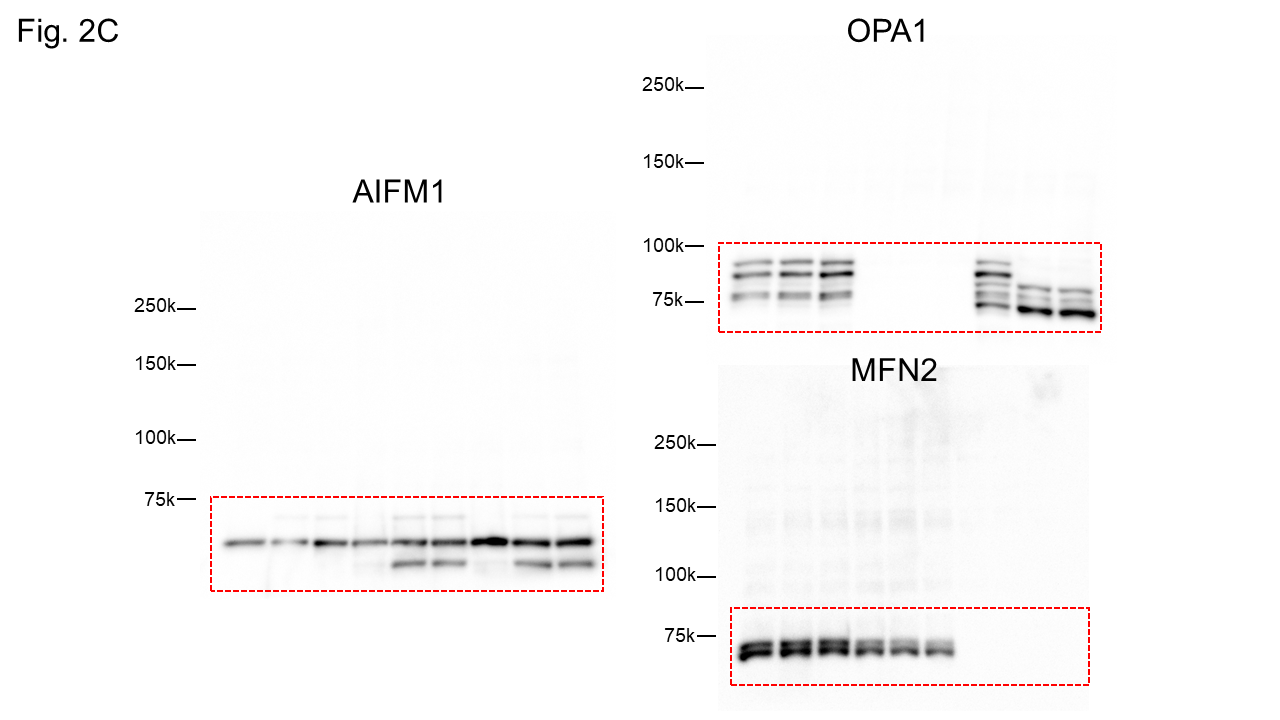

Supplement: Supplementary file 10 — Source data Fig. 2 [file 44318_2026_734_MOESM10_ESM.zip › Source Data Figure 2/2C/2C_blot.TIF]

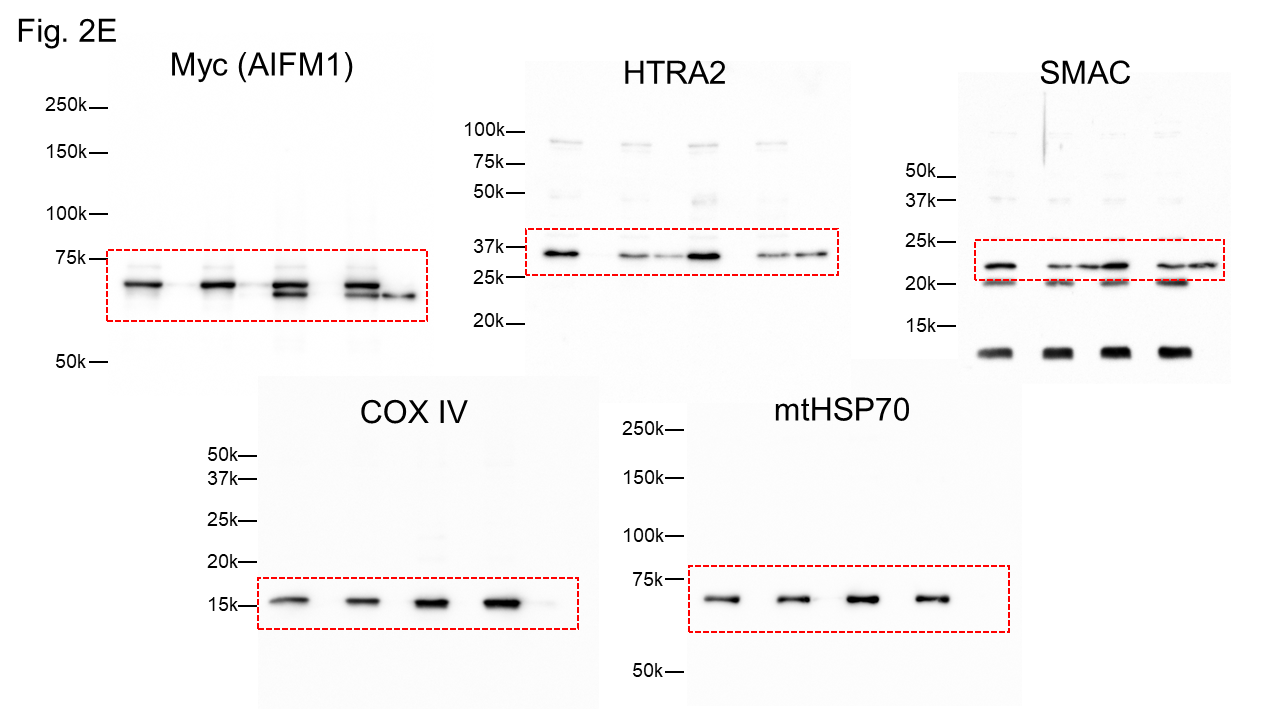

Supplement: Supplementary file 10 — Source data Fig. 2 [file 44318_2026_734_MOESM10_ESM.zip › Source Data Figure 2/2E/2E_blot.TIF]

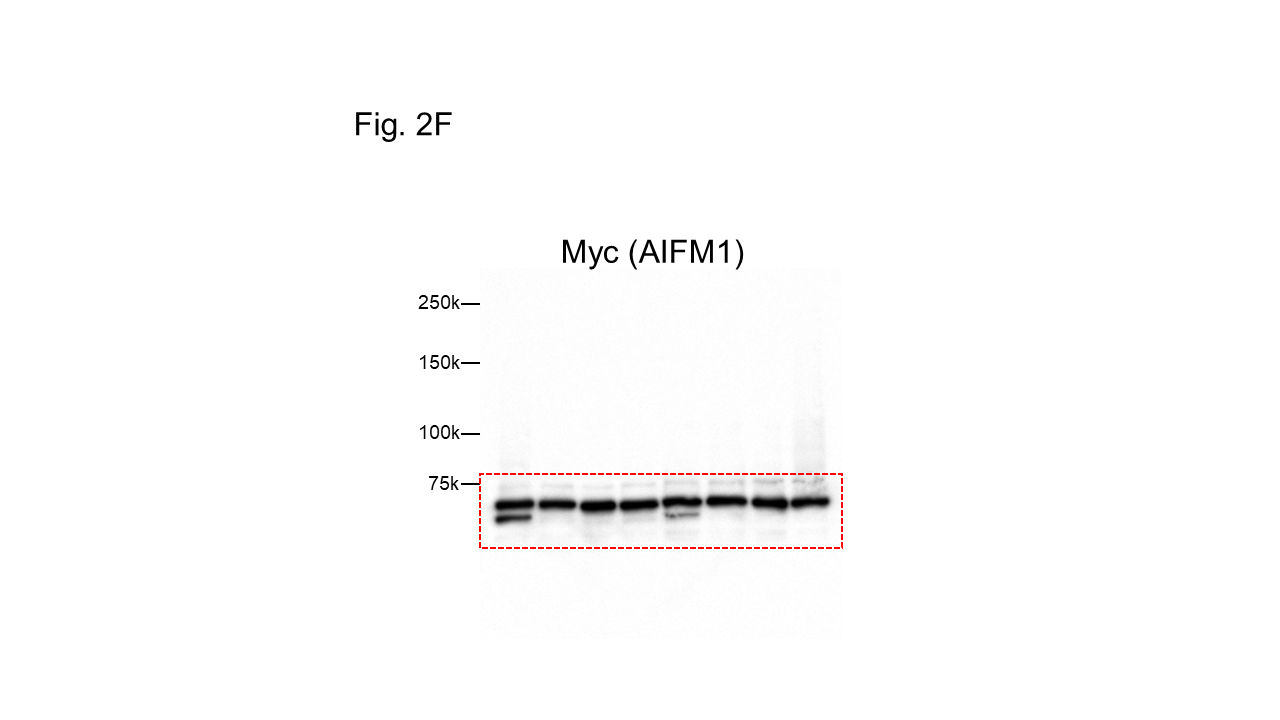

Supplement: Supplementary file 10 — Source data Fig. 2 [file 44318_2026_734_MOESM10_ESM.zip › Source Data Figure 2/2F/2F_blot.tif]

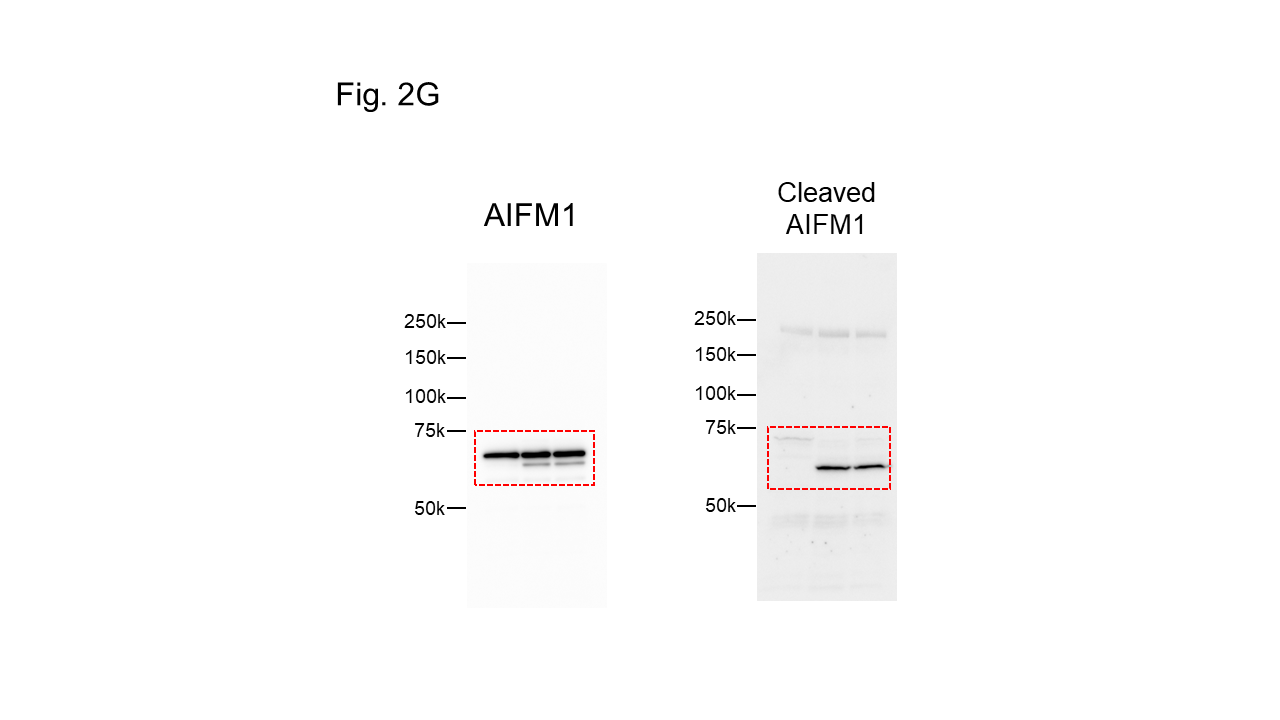

Supplement: Supplementary file 10 — Source data Fig. 2 [file 44318_2026_734_MOESM10_ESM.zip › Source Data Figure 2/2G/2G_blot.tif]

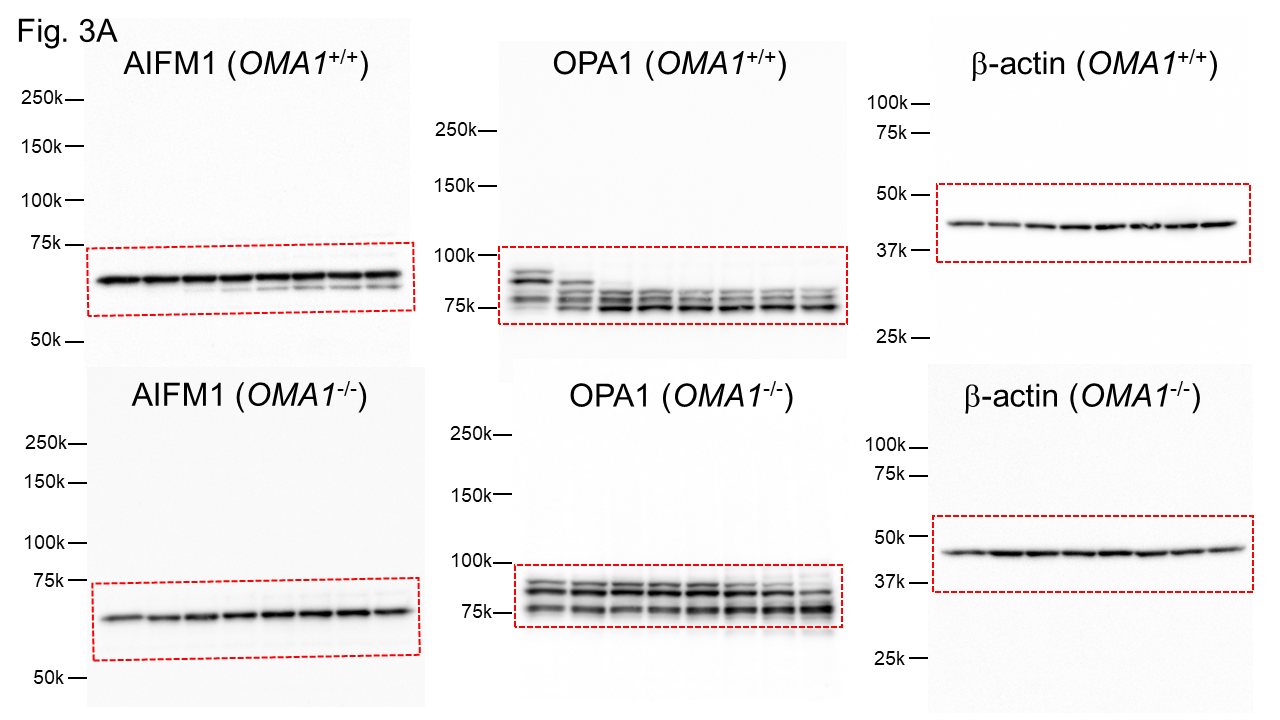

Supplement: Supplementary file 11 — Source data Fig. 3 [file 44318_2026_734_MOESM11_ESM.zip › Source Data Figure 3/3A/3A_blot.TIF]

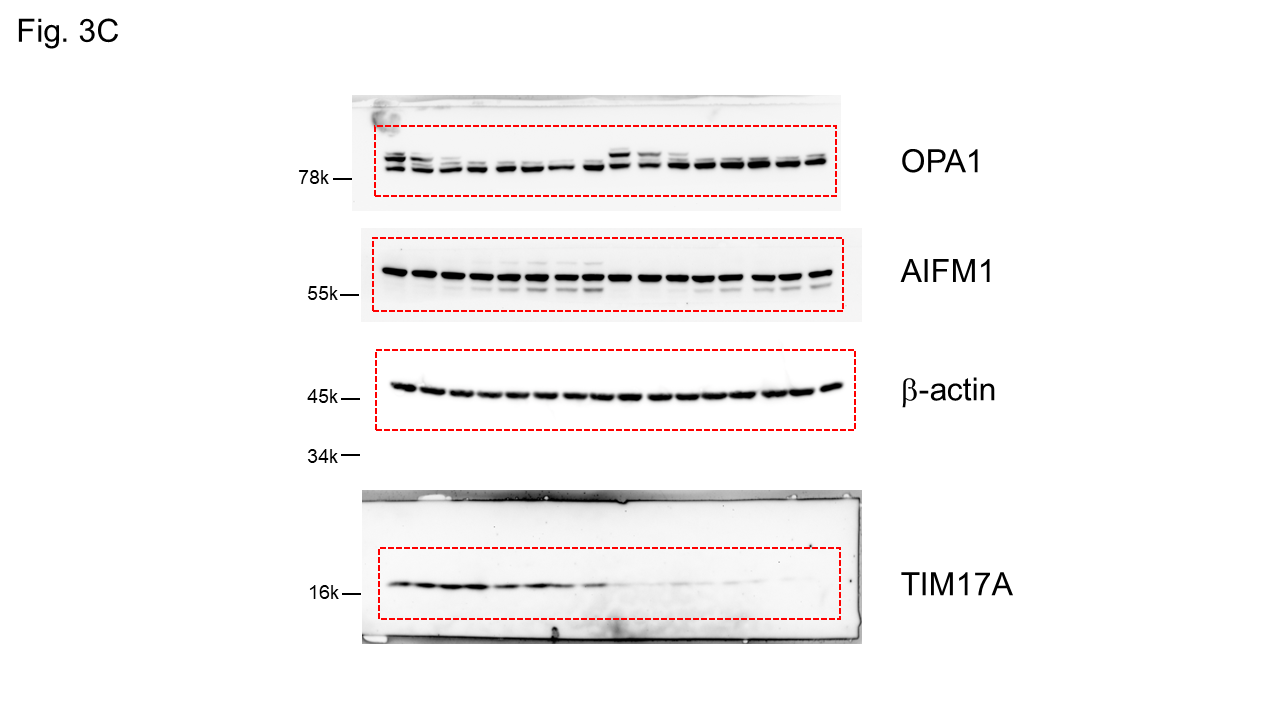

Supplement: Supplementary file 11 — Source data Fig. 3 [file 44318_2026_734_MOESM11_ESM.zip › Source Data Figure 3/3C/3C_blot.TIF]

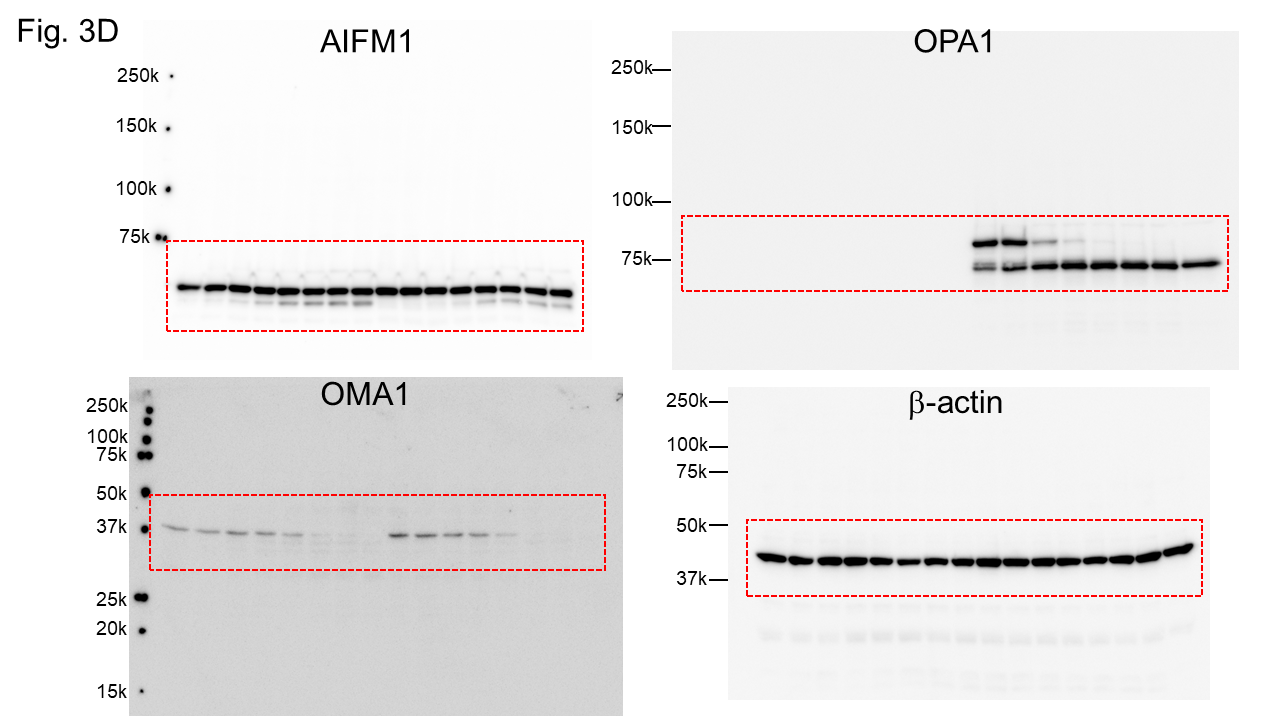

Supplement: Supplementary file 11 — Source data Fig. 3 [file 44318_2026_734_MOESM11_ESM.zip › Source Data Figure 3/3D/3D_blot.TIF]

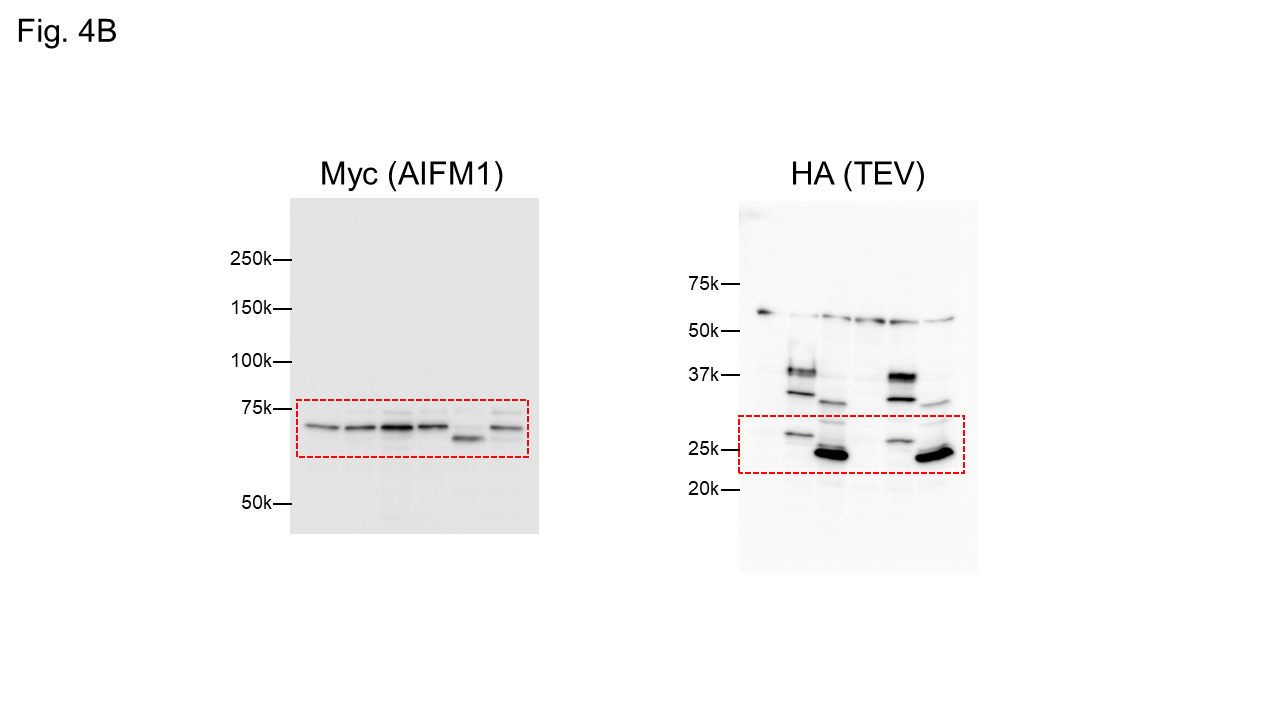

Supplement: Supplementary file 12 — Source data Fig. 4 [file 44318_2026_734_MOESM12_ESM.zip › Source Data Figure 4/4B/4B_blot.TIF]

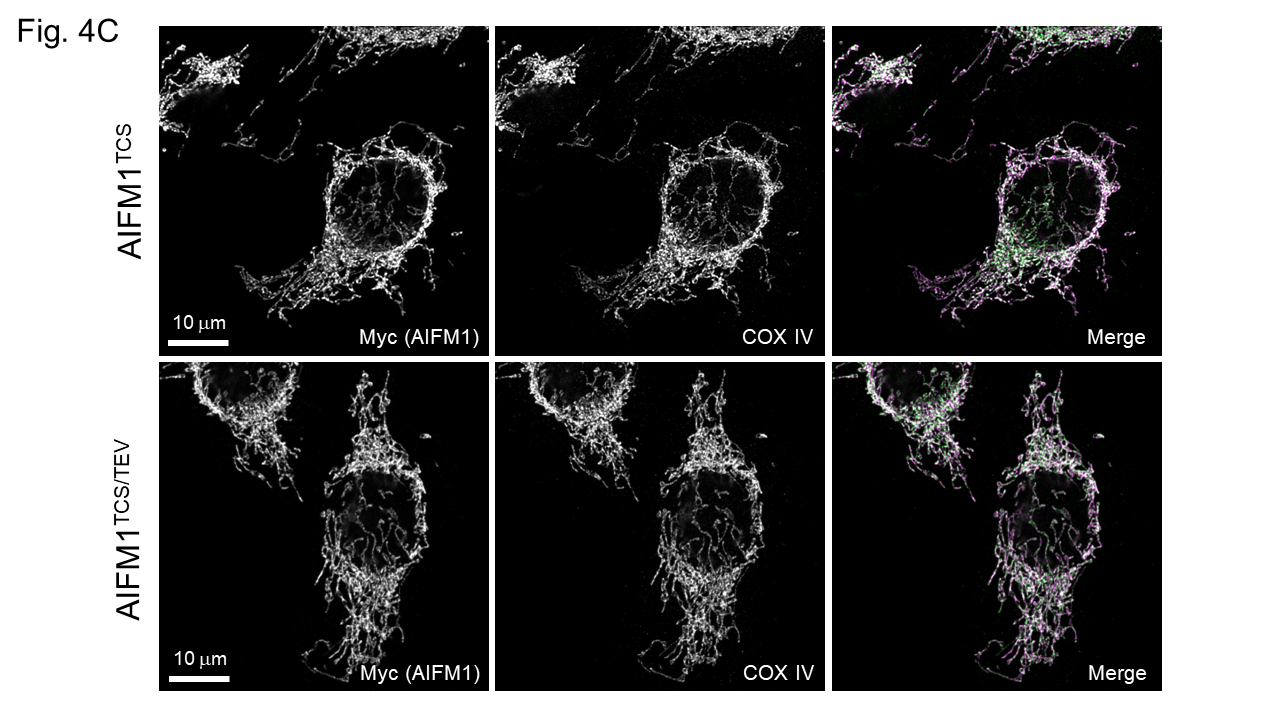

Supplement: Supplementary file 12 — Source data Fig. 4 [file 44318_2026_734_MOESM12_ESM.zip › Source Data Figure 4/4C/4C_image.TIF]

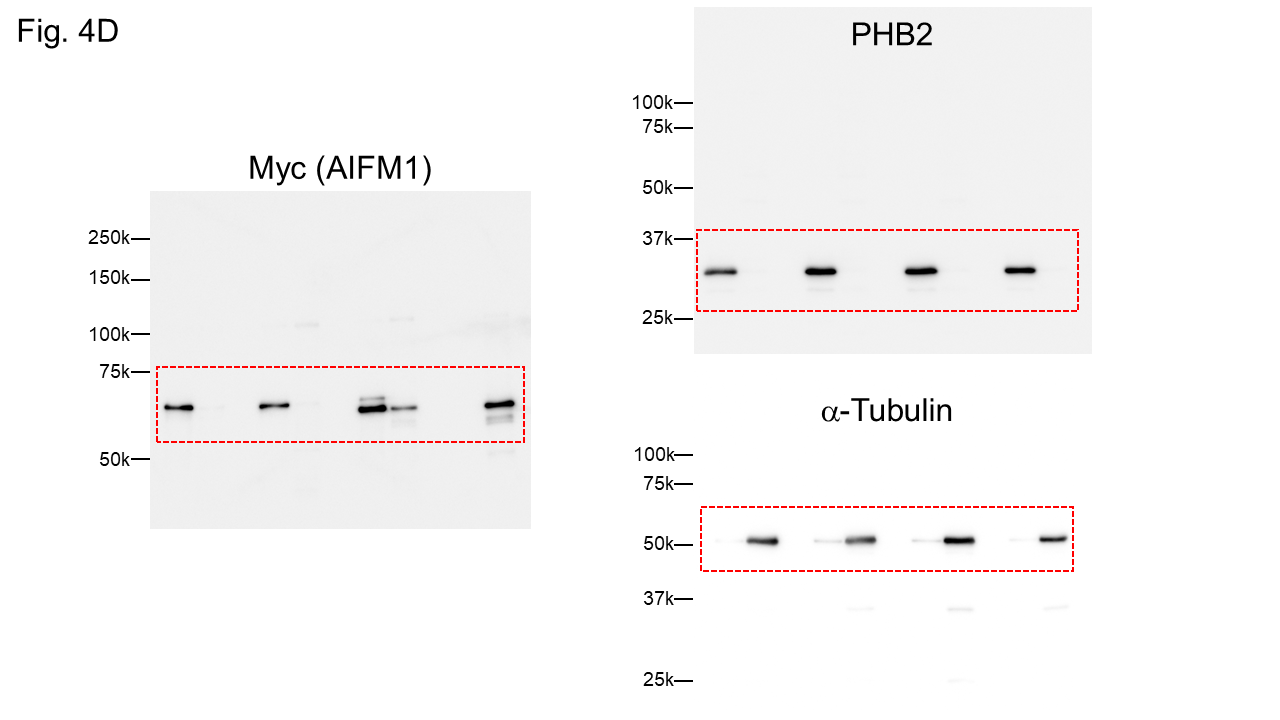

Supplement: Supplementary file 12 — Source data Fig. 4 [file 44318_2026_734_MOESM12_ESM.zip › Source Data Figure 4/4D/4D_blot.TIF]

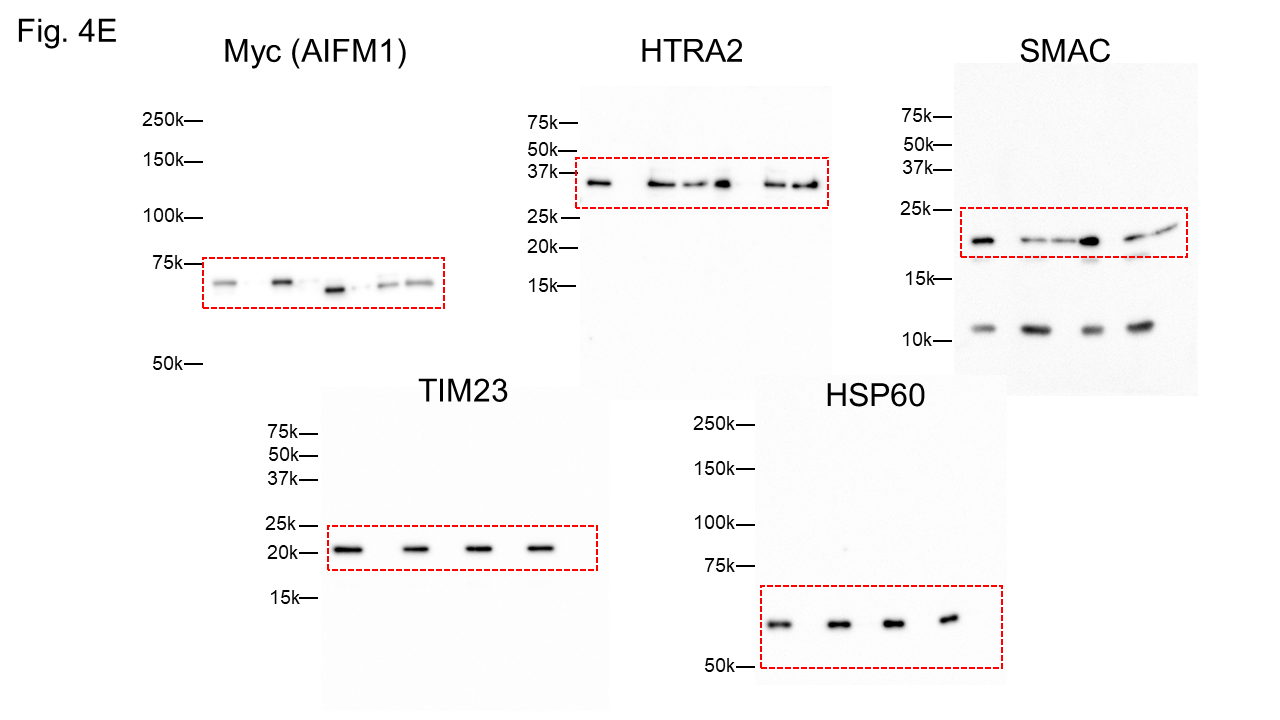

Supplement: Supplementary file 12 — Source data Fig. 4 [file 44318_2026_734_MOESM12_ESM.zip › Source Data Figure 4/4E/4E_blot.TIF]

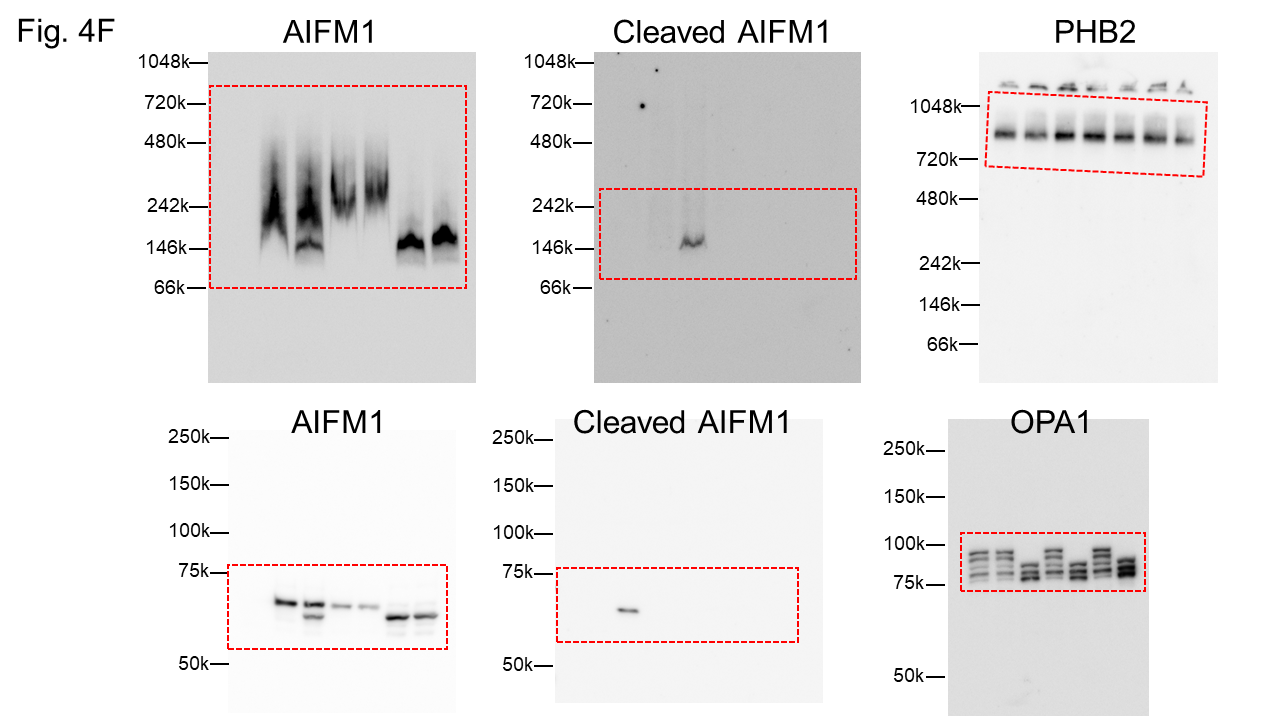

Supplement: Supplementary file 12 — Source data Fig. 4 [file 44318_2026_734_MOESM12_ESM.zip › Source Data Figure 4/4F/4F_blot.TIF]

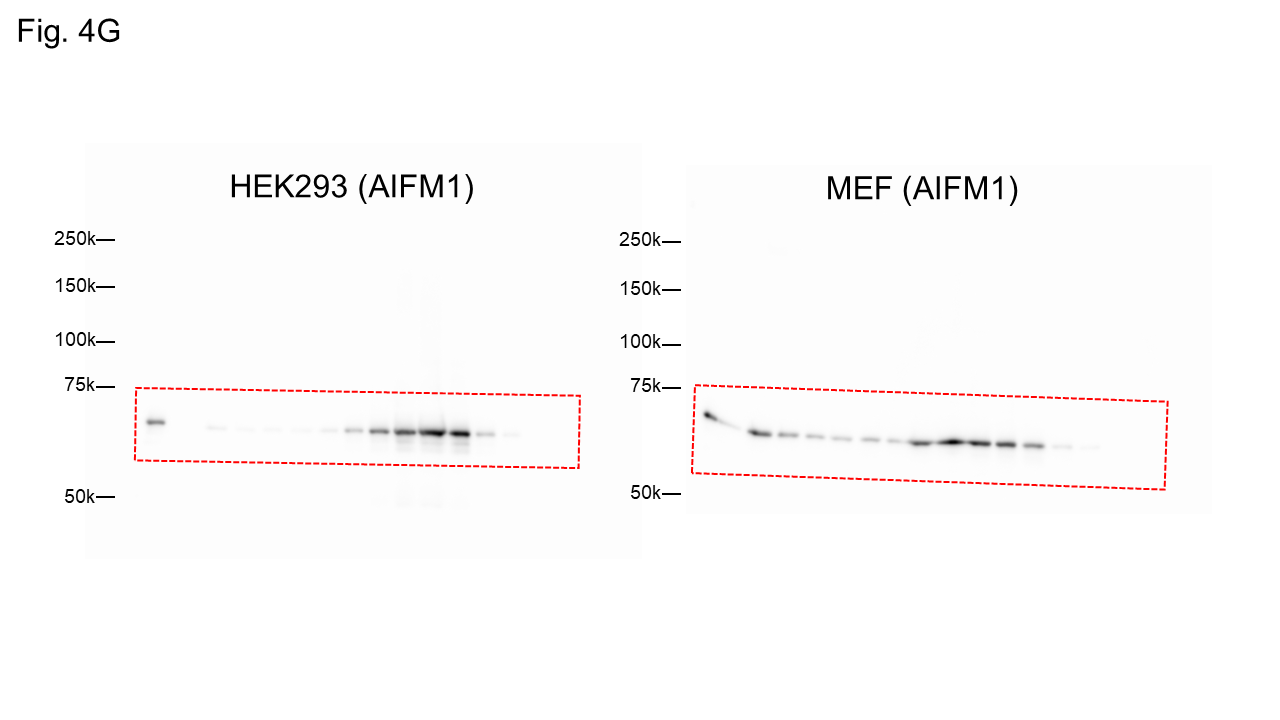

Supplement: Supplementary file 12 — Source data Fig. 4 [file 44318_2026_734_MOESM12_ESM.zip › Source Data Figure 4/4G/4G_blot.TIF]

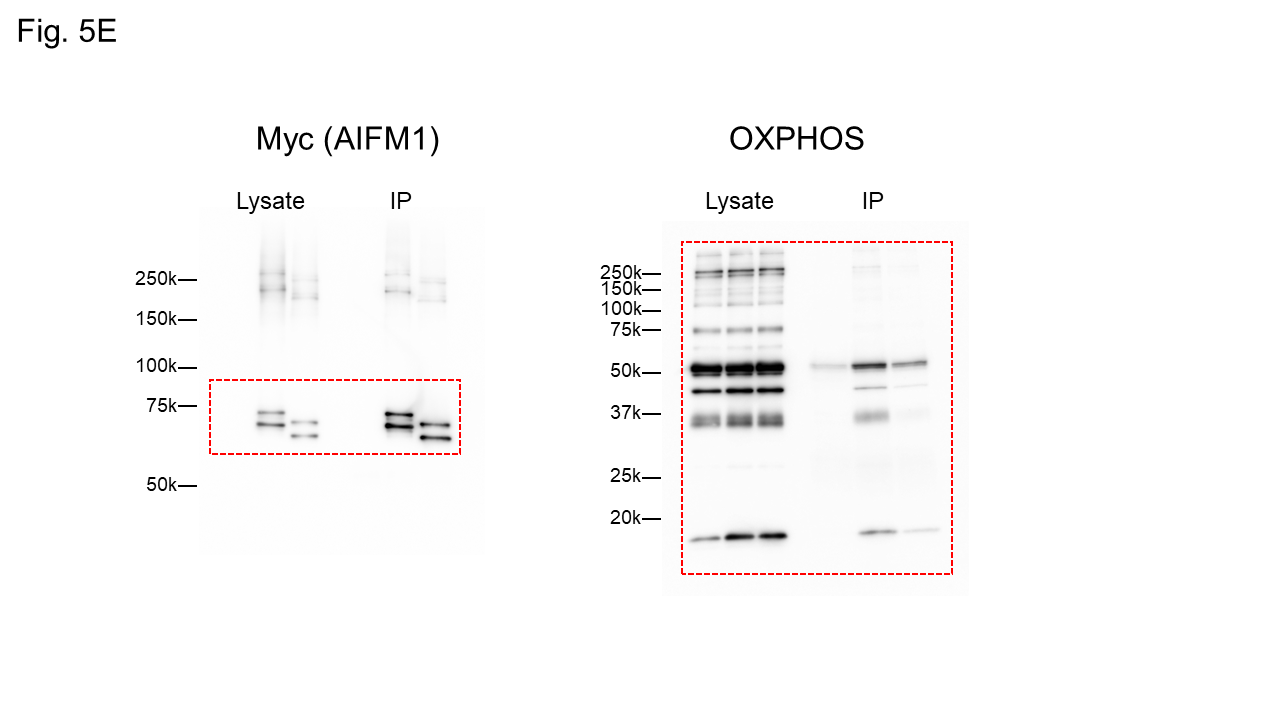

Supplement: Supplementary file 13 — Source data Fig. 5 [file 44318_2026_734_MOESM13_ESM.zip › Source Data Figure 5/5E/5E_blot.TIF]

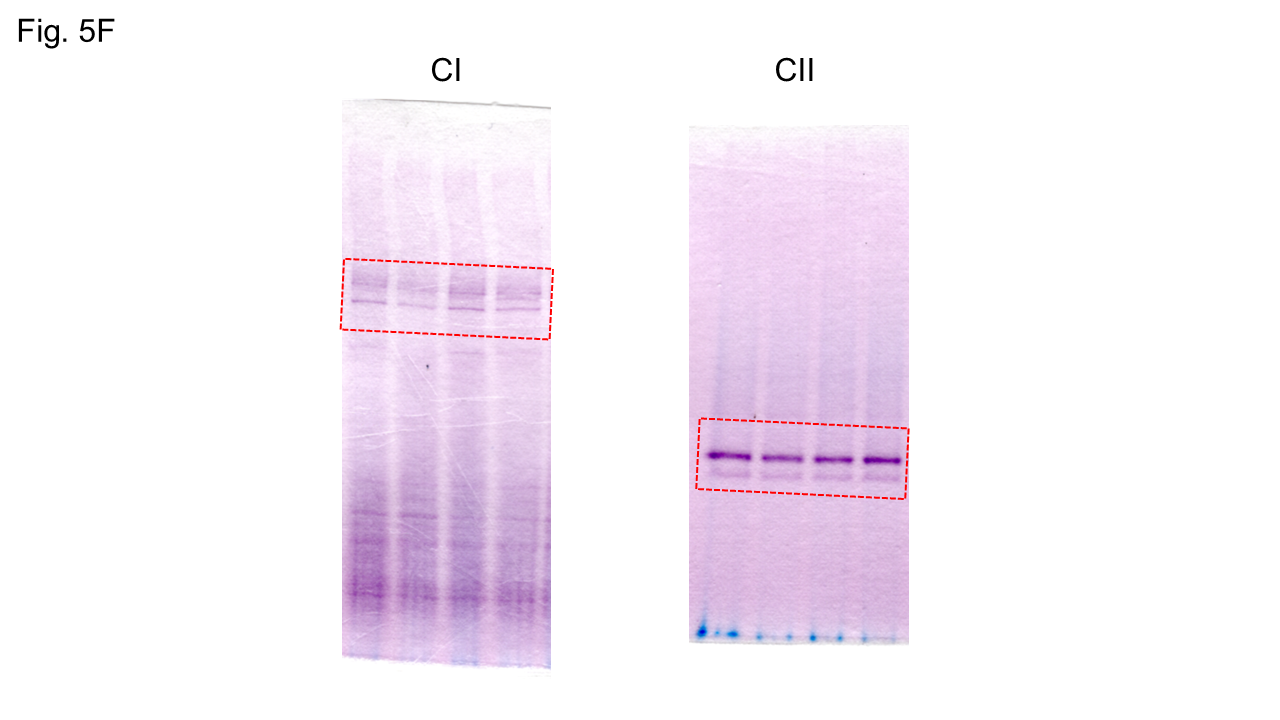

Supplement: Supplementary file 13 — Source data Fig. 5 [file 44318_2026_734_MOESM13_ESM.zip › Source Data Figure 5/5F/5F_stain.TIF]

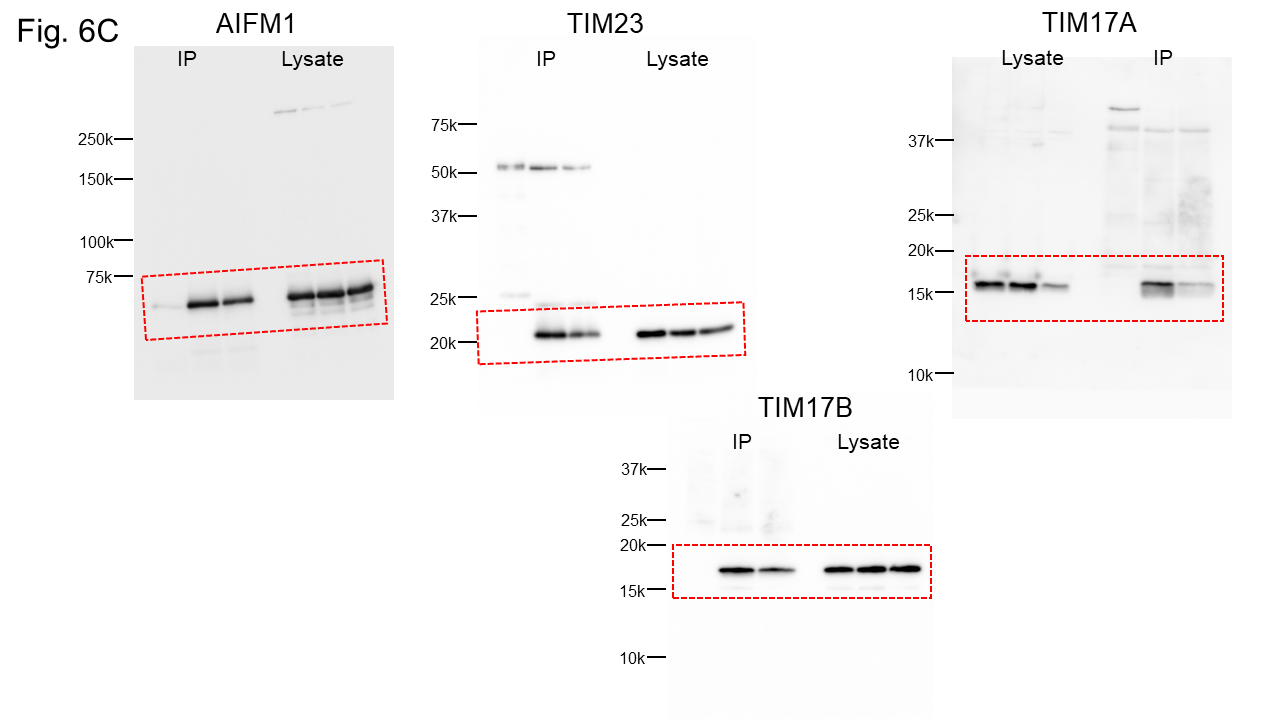

Supplement: Supplementary file 14 — Source data Fig. 6 [file 44318_2026_734_MOESM14_ESM.zip › Source Data Figure 6/6C/6C_blot.TIF]

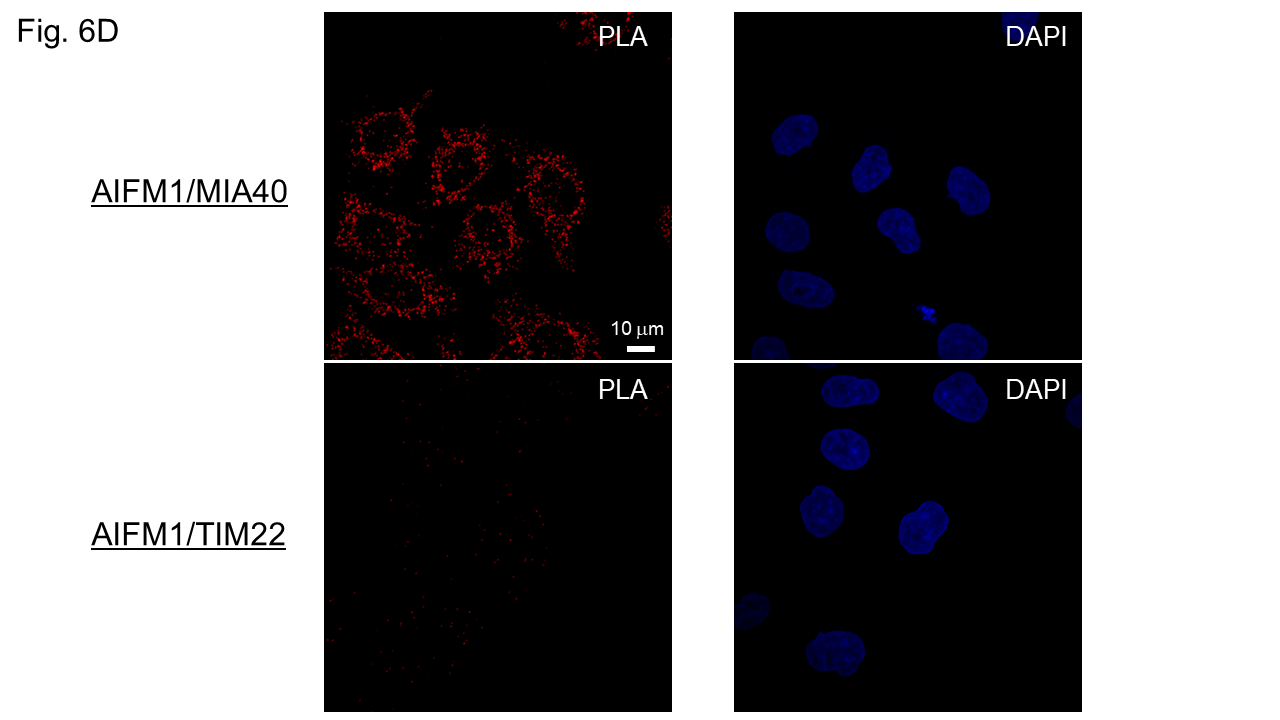

Supplement: Supplementary file 14 — Source data Fig. 6 [file 44318_2026_734_MOESM14_ESM.zip › Source Data Figure 6/6D/6D_image AIFM1_MIA40.TIF]

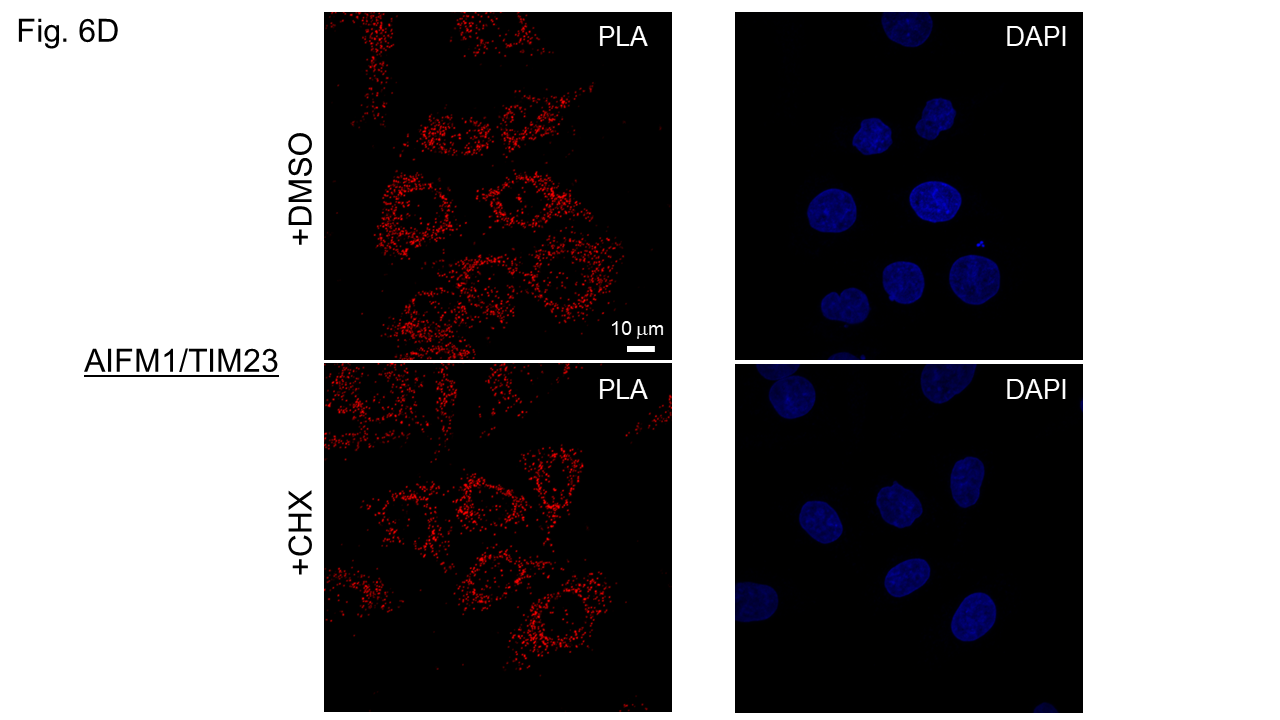

Supplement: Supplementary file 14 — Source data Fig. 6 [file 44318_2026_734_MOESM14_ESM.zip › Source Data Figure 6/6D/6D_image AIFM1_TIM23.TIF]

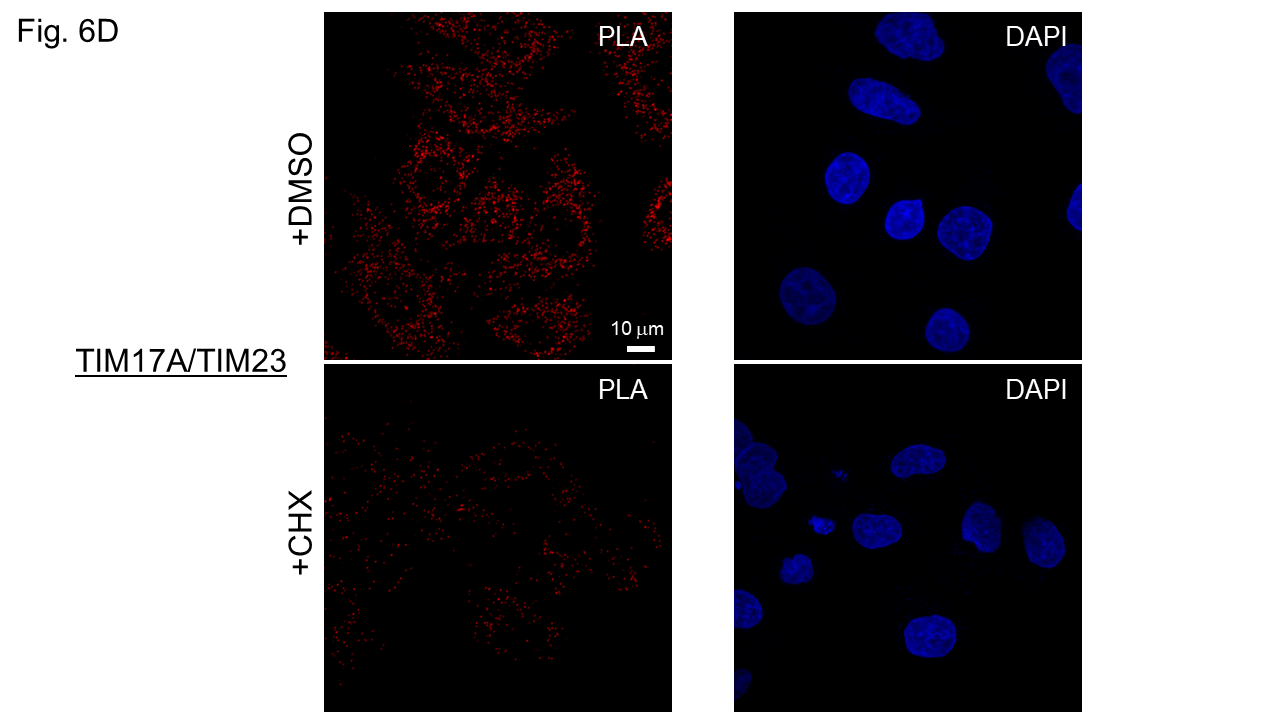

Supplement: Supplementary file 14 — Source data Fig. 6 [file 44318_2026_734_MOESM14_ESM.zip › Source Data Figure 6/6D/6D_image TIM17A_TIM23.TIF]

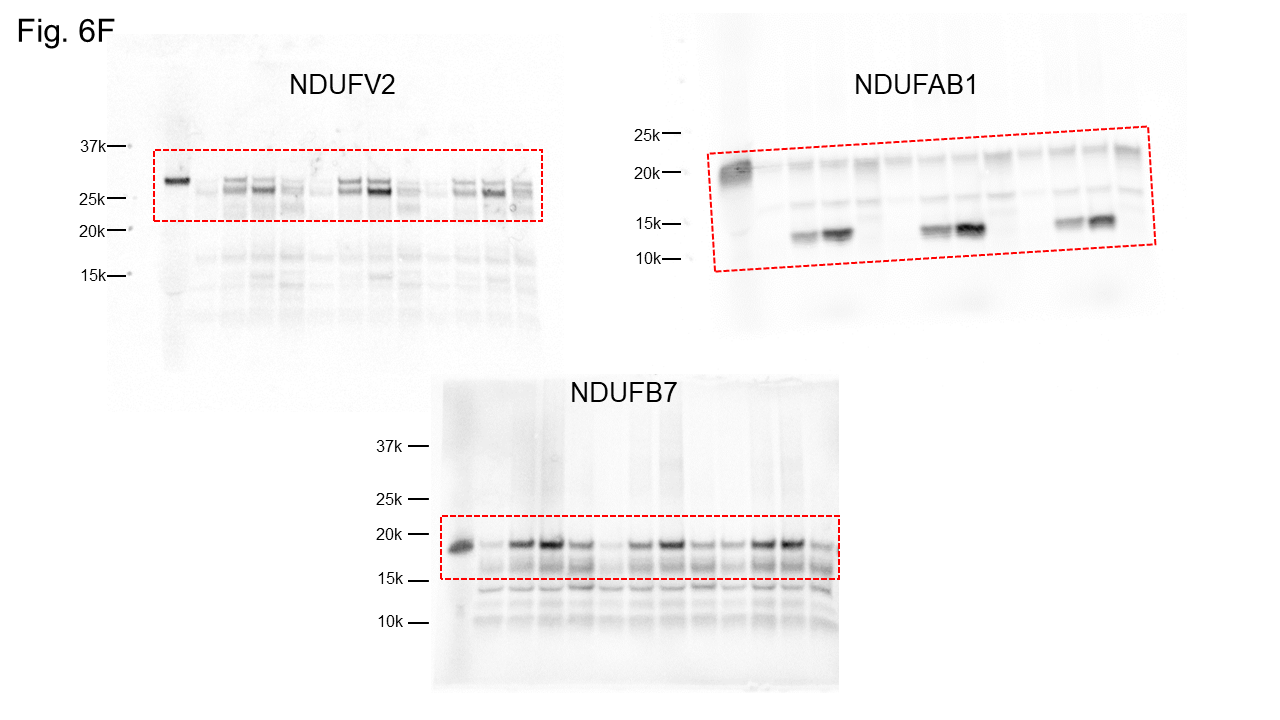

Supplement: Supplementary file 14 — Source data Fig. 6 [file 44318_2026_734_MOESM14_ESM.zip › Source Data Figure 6/6F/6F_RI.TIF]

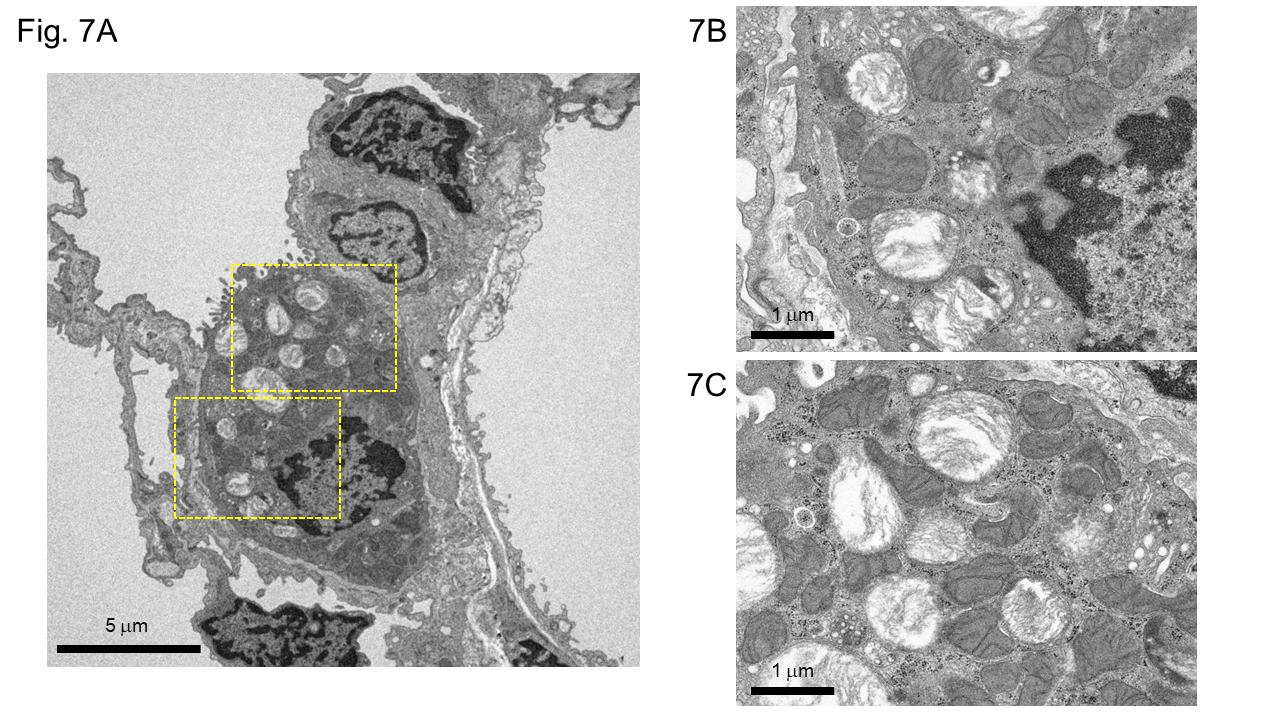

Supplement: Supplementary file 15 — Source data Fig. 7 [file 44318_2026_734_MOESM15_ESM.zip › Source Data Figure 7/7A-C/7A, 7B, 7C_TEM.TIF]

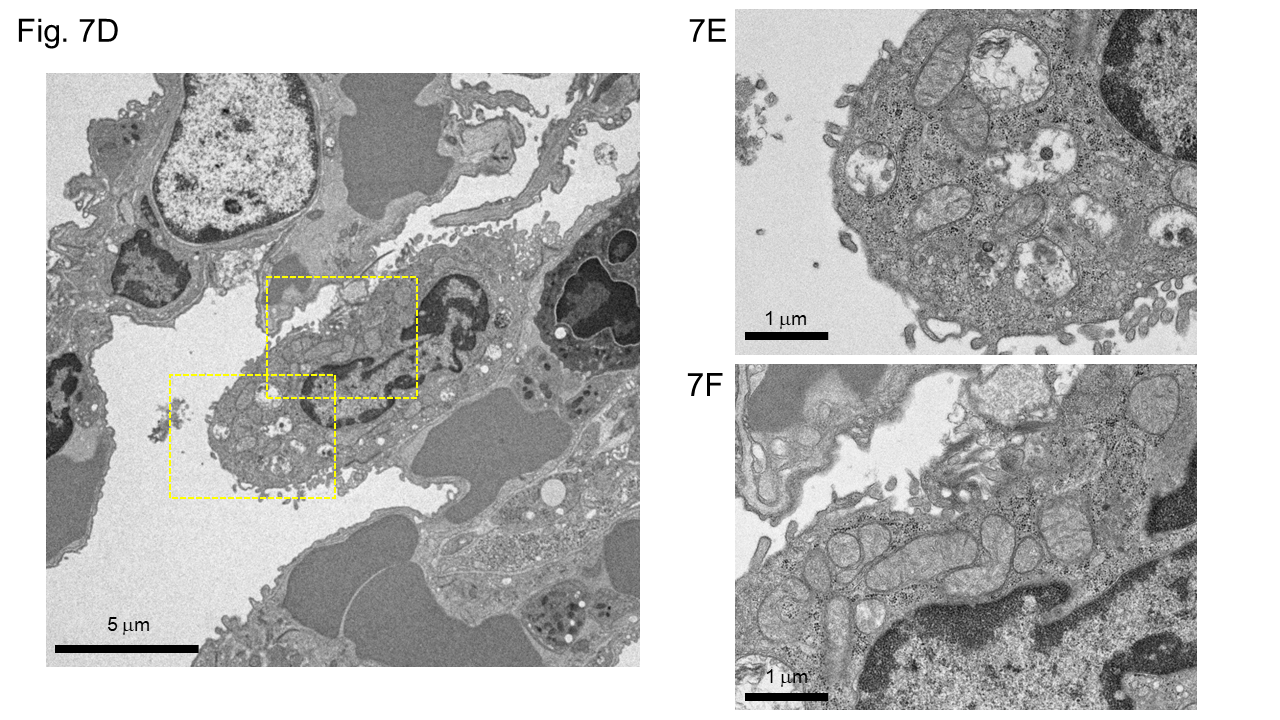

Supplement: Supplementary file 15 — Source data Fig. 7 [file 44318_2026_734_MOESM15_ESM.zip › Source Data Figure 7/7D-F/7D, 7E, 7F_TEM.TIF]

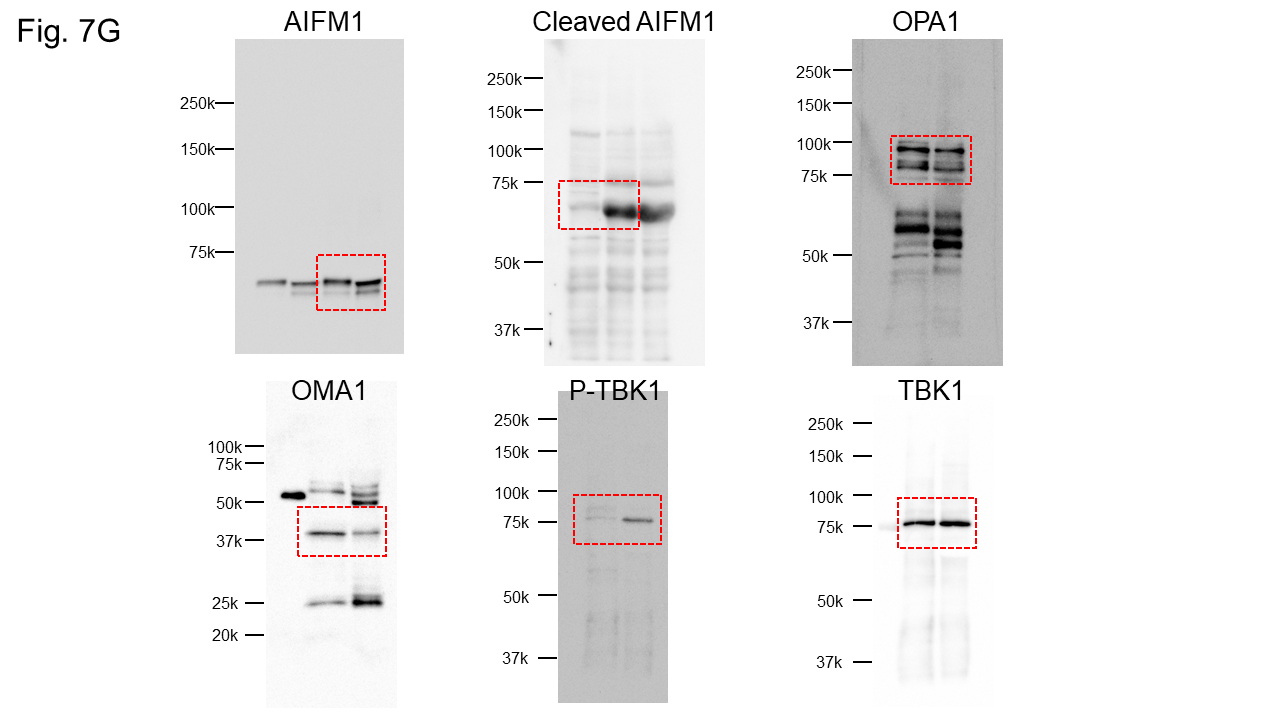

Supplement: Supplementary file 15 — Source data Fig. 7 [file 44318_2026_734_MOESM15_ESM.zip › Source Data Figure 7/7G/7G_blot.TIF]
