# Supplementary material for: Stress-induced OMA1-mediated cleavage of AIFM1 suppresses cell growth by controlling mitochondrial OXPHOS activity
Source: EMBO J. 2026 Mar 24;45(11):3655–98. doi: 10.1038/s44318-026-00734-y (PMC13226697; doi:10.1038/s44318-026-00734-y)
Supplement: Supplementary file 16 — Expanded View Figures [file 44318_2026_734_MOESM16_ESM.pdf]

Expanded View Figures

Figure EV1. Stress-inducible AIFM1 processing by OMA1.

(**A**) *OMA1*$^{-/-}$ MEFs stably expressing OMA1/Myc or OMA1^{E324Q}/Myc were treated for 3 h with either FCCP (40 μM) or valinomycin (1 μg/mL) and analyzed by immunoblotting with the indicated antibodies. OMA1^{E324Q}/Myc accumulated stably in cells upon the loss of $\Delta\Psi_m$ (bottom two right lanes), whereas OMA1/Myc was rapidly degraded by autocatalytic proteolysis. (**B**) Stress-induced substrates (AIFM1 and OPA1) processed by OMA1. *OMA1*$^{+/+}$ (WT) and *OMA1*$^{-/-}$ MEFs were incubated for 3 h with FCCP, H_2O_2, rotenone, antimycin A (Anti A), oligomycin A (Oligo A), or Anti A/Oligo A and analyzed by western blotting. (**C**) AIFM1 processing levels observed in *Cox10*$^{-/-}$ hearts (arrow) were notably reduced in *Cox10*$^{-/-}$*OMA1*$^{-/-}$ hearts ($n = 2$). (**D**) *OMA1*$^{-/-}$*YME1L*$^{-/-}$ (DKO) MEFs stably expressing either OMA1/Myc or YME1L/HA were incubated for 3 h in the absence (DMSO) or presence of either FCCP (40 μM) or valinomycin (1 μg/mL) and analyzed by immunoblotting (indicated antibodies). (**E**) OMA1-dependent processing of AIFM1 in *Drp1 KO* cells. The WT or *Drp1* KO MEFs were treated for 3 h with either FCCP (40 μM) or valinomycin (1 μg/mL) and analyzed by immunoblotting with the indicated antibodies. (**F**) Illustration shows a hypothetical model of OMA1-mediated AIFM1 processing on the same membrane (left, in a dotted box, acting in *cis*) or on different membranes (right, acting in *trans*). *Mfns*-DKO and *OPA1*-KO are incompetent for OM and IM fusion, respectively. *Drp1*-KO is incompetent for mitochondrial fission.

© The Author(s)

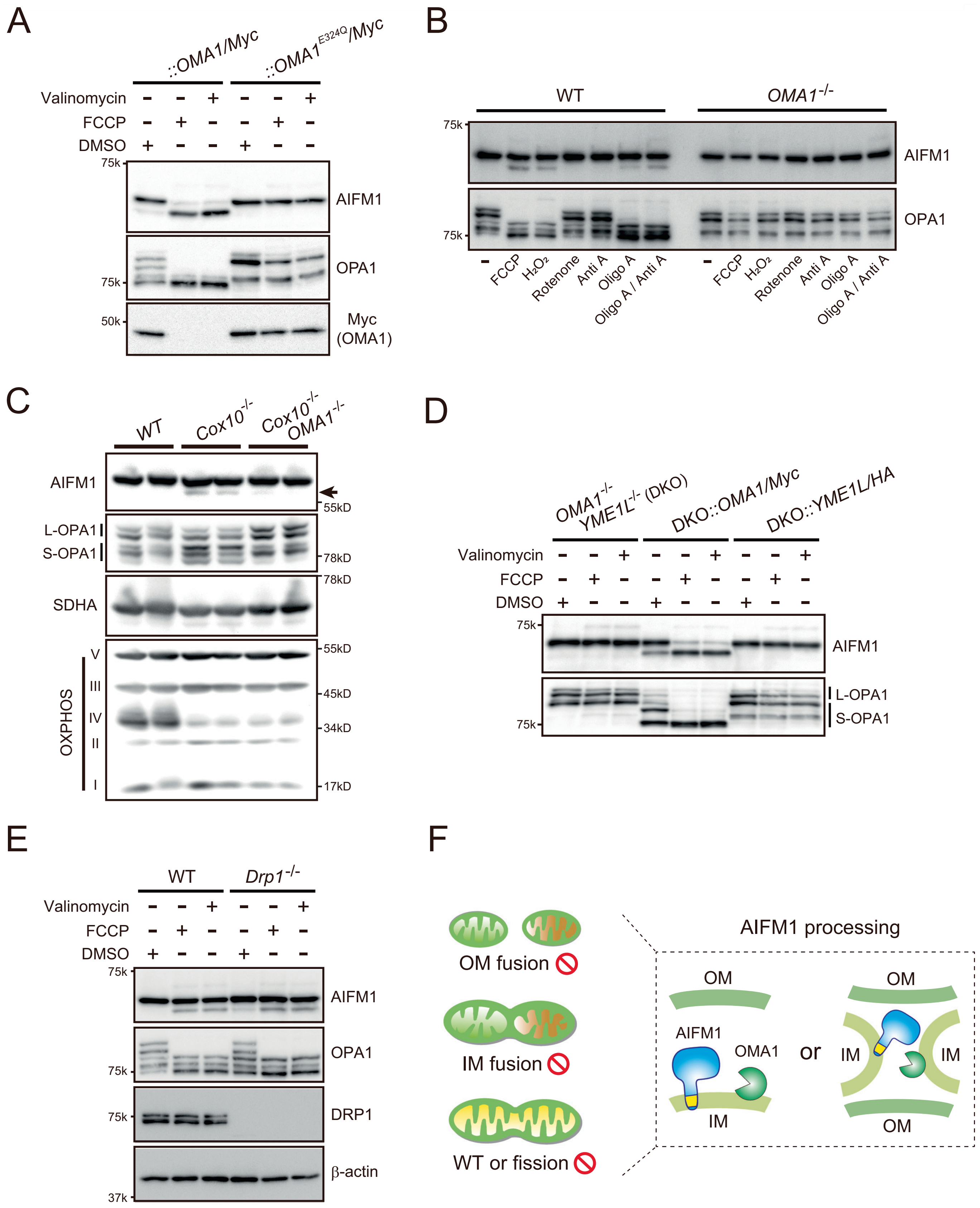
A
::OMA1/Myc
::OMA1E324Q/Myc
Valinomycin - - + - - +
FCCP - + - - + -
DMSO + - - + - -
75k
AIFM1
OPA1
75k
50k
Myc (OMA1)
B
WT
OMA1-/-
75k
AIFM1
OPA1
75k
- FCCP H2O2 Rotenone Anti A Oligo A Oligo A / Anti A
- FCCP H2O2 Rotenone Anti A Oligo A Oligo A / Anti A
C
WT
Cox10-/-
Cox10-/- OMA1-/-
AIFM1
55kD
L-OPA1
S-OPA1
78kD
78kD
SDHA
55kD
V
III
45kD
IV
34kD
II
I
17kD
OXPHOS
D
OMA1-/- YME1L-/- (DKO)
DKO::OMA1/Myc
DKO::YME1L/HA
Valinomycin - - + - - + - - +
FCCP - + - - + - - + -
DMSO + - - + - - + - -
75k
AIFM1
L-OPA1
S-OPA1
75k
E
WT
Drp1-/-
Valinomycin - - + - - +
FCCP - + - - + -
DMSO + - - + - -
75k
AIFM1
OPA1
75k
75k
DRP1
β-actin
37k
F
OM fusion
IM fusion
WT or fission
AIFM1 processing
OM
AIFM1
OMA1
IM
or
OM
IM
IM
OM

 © The Author(s)

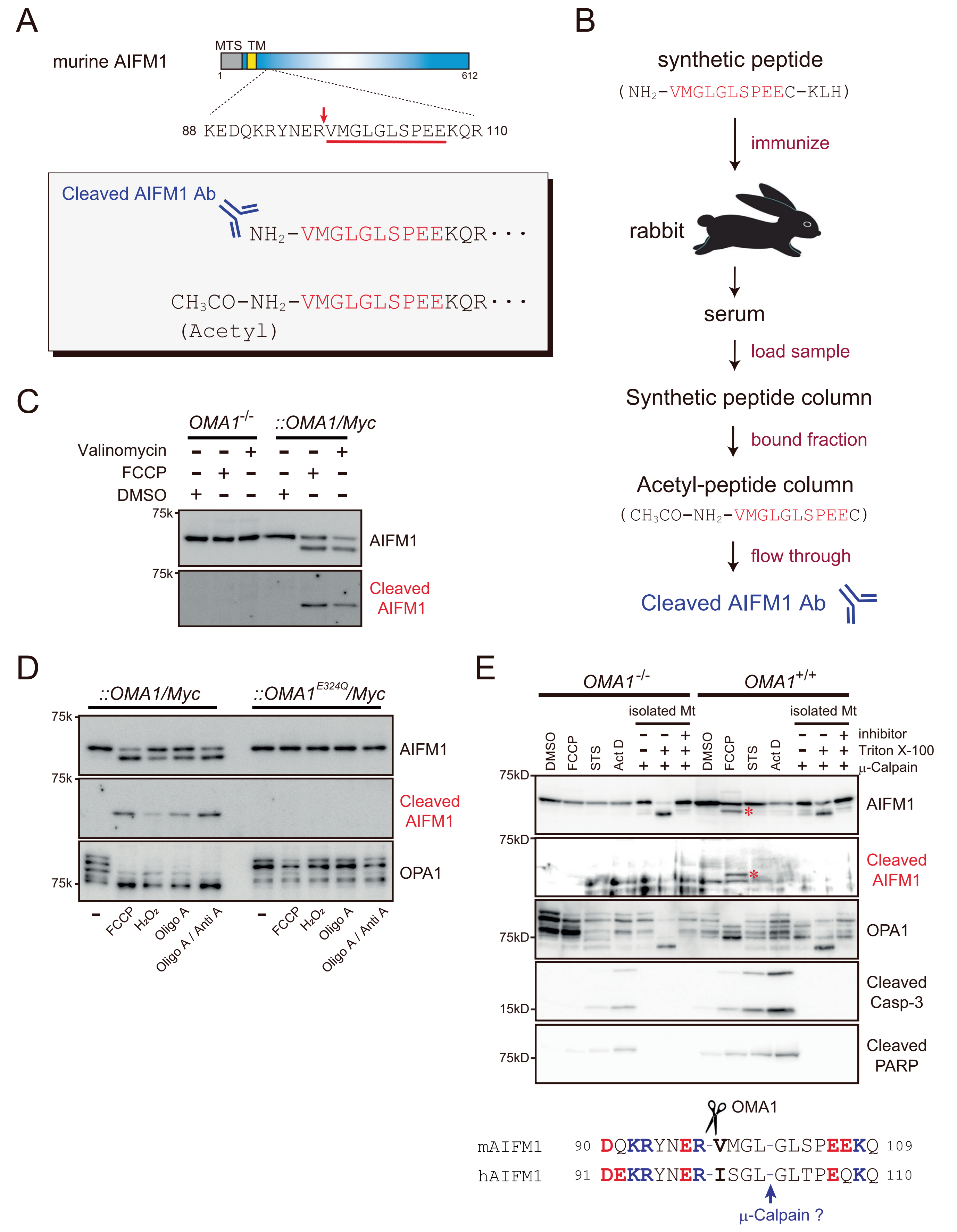
A
murine AIFM1
MTS TM
1
612
88 KEDQKRYNERVMGLGLSPEEKQR 110
Cleaved AIFM1 Ab
NH2-VMGLGLSPEEKQR···
CH3CO-NH2-VMGLGLSPEEKQR···
(Acetyl)
B
synthetic peptide
(NH2-VMGLGLSPEEC-KLH)
immunize
rabbit
serum
load sample
Synthetic peptide column
bound fraction
Acetyl-peptide column
(CH3CO-NH2-VMGLGLSPEEC)
flow through
Cleaved AIFM1 Ab
C
OMA1-/-
::OMA1/Myc
Valinomycin
FCCP
DMSO
75k
AIFM1
75k
Cleaved AIFM1
D
::OMA1/Myc
::OMA1E324Q/Myc
75k
AIFM1
75k
Cleaved AIFM1
OPA1
75k
FCCP
H2O2
Oligo A
Oligo A / Anti A
E
OMA1-/-
OMA1+/+
isolated Mt
DMSO
FCCP
STS
Act D
inhibitor
Triton X-100
μ-Calpain
75kD
AIFM1
75kD
Cleaved AIFM1
75kD
OPA1
15kD
Cleaved Casp-3
75kD
Cleaved PARP
OMA1
mAIFM1 90 DQKRYNER-VMGL-GLSPEEKQ 109
hAIFM1 91 DEKRYNER-ISGL-GLTPEQKQ 110
μ-Calpain ?

Figure EV2. Scheme for generating an N-terminal specific antibody against AIFM1.

(**A**) Position of the AIFM1 cleavage site (red arrow) targeted by OMA1. The sequence portion underlined in red was chemically synthesized for immunized rabbits to generate polyclonal antibodies against the region (lower inset, top). The same N-terminally acetylated peptide (lower inset, bottom) was also synthesized for use in affinity purification. (**B**) Flowchart of the generation of the custom antibody against the N-terminal portion of AIFM1. (**C**, **D**) *OMA1*$^{-/-}$ or *OMA1*$^{-/-}$ MEFs stably expressing OMA1/Myc were treated for 3 h with either FCCP (40 μM) or valinomycin (1 μg/mL) and analyzed by immunoblotting with the custom cleaved AIFM1 antibody (bottom). The antibody specificity was confirmed by comparing the same immunoblot with that detected by the normal AIFM1 antibody (top). In (**D**), *OMA1*$^{-/-}$ MEFs stably expressing OMA1/Myc or OMA1^{E324Q}/Myc were treated for 3 h with the indicated chemicals and analyzed by immunoblotting with the indicated antibodies. (**E**) *OMA1*$^{+/+}$ and *OMA1*$^{-/-}$ MEFs were treated with FCCP (20 μM), staurosporine (STS, 1 μM), or actinomycin D (Act D, 20 μM) for 12 h to induce cell death. The cells were then analyzed by immunoblotting with the indicated antibodies. In addition to the experiment involving cells treated with proapoptotic drugs, mitochondria were isolated from *OMA1*$^{+/+}$ and *OMA1*$^{-/-}$ MEFs, and then incubated for 30 min at 25 °C with μ-calpain, with or without Triton X-100 (0.1%) and a calpain I inhibitor. AIFM1 processing was not observed under these conditions during apoptotic stimuli, whereas μ-calpain-mediated AIFM1 cleavage was OMA1-independent, and the cleavage sites were distinct (see red asterisk). The bottom sequences indicate the AIFM1 cleavage sites by OMA1 and by μ-calpain, as previously reported (Polster et al, 2005).

 © The Author(s)

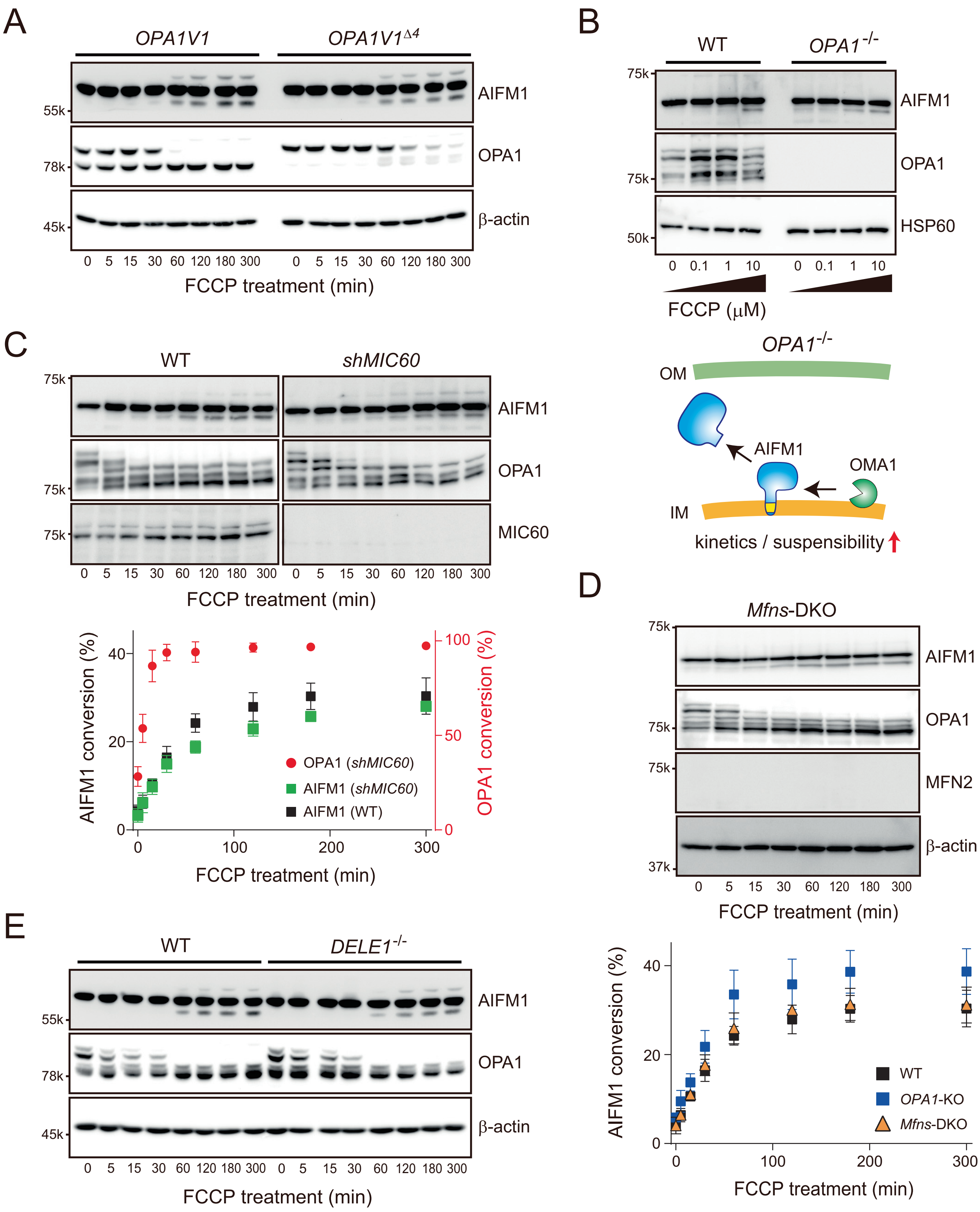
A
OPA1V1
OPA1V1Δ4
AIFM1
OPA1
β-actin
55k
78k
45k
0 5 15 30 60 120 180 300 0 5 15 30 60 120 180 300
FCCP treatment (min)
B
WT
OPA1-/-
75k
75k
50k
AIFM1
OPA1
HSP60
0 0.1 1 10 0 0.1 1 10
FCCP (μM)
OPA1-/-
OM
AIFM1
OMA1
IM
kinetics / suspensibility
C
WT
shMIC60
75k
75k
75k
AIFM1
OPA1
MIC60
0 5 15 30 60 120 180 300 0 5 15 30 60 120 180 300
FCCP treatment (min)
AIFM1 conversion (%)
OPA1 conversion (%)
OPA1 (shMIC60)
AIFM1 (shMIC60)
AIFM1 (WT)
FCCP treatment (min)
D
Mfns-DKO
75k
75k
75k
37k
AIFM1
OPA1
MFN2
β-actin
0 5 15 30 60 120 180 300
FCCP treatment (min)
AIFM1 conversion (%)
WT
OPA1-KO
Mfns-DKO
FCCP treatment (min)
E
WT
DELE1-/-
AIFM1
OPA1
β-actin
55k
78k
45k
0 5 15 30 60 120 180 300 0 5 15 30 60 120 180 300
FCCP treatment (min)

Figure EV3. Role of OPA1 in OMA1-dependent AIFM1 processing.

(**A**) MEFs only stably expressing either the OPA1 V1 isoform (*OPA1V1*) or its non-cleavable version (*OPA1V1*$^{\Delta4}$) (Ahola et al, 2024) incubated with FCCP (40 μM) were collected at the indicated time points (0, 5, 15, 30, 60, 120, 180, and 300 min) and analyzed by western blot. β-Actin blots were used as loading controls for each time point. (**B**) WT or *OPA1*$^{-/-}$ MEFs were treated for 3 h with different concentrations of FCCP (0, 0.1, 1 and 10 μM) and analyzed by immunoblotting with the indicated antibodies. Bottom illustration, Model of OMA1-mediated AIFM1 processing in the absence of OPA1. The loss of OPA1 increases susceptibility to and accelerates the attack of AIFM1 by OMA1. (**C**) MEFs without or with expression of shRNA against *MIC60* incubated with FCCP (40 μM) were collected at the indicated time points (0, 5, 15, 30, 60, 120, 180, and 300 min) and analyzed by western blot. Bottom graph, OMA1-dependent substrate processing in *shMIC60* MEFs (red, OPA1; green, AIFM1) was quantified ($n = 3$ biologic replicates) and their conversions (%) were plotted (mean values ± SD). AIFM1 processing in WT MEFs (control) is shown in black. (**D**) Similar to (**C**), except that the *Mfns*-DKO MEFs were treated with FCCP (40 μM) for the indicated times. The bottom graph shows the quantitative data (mean values ± SD) obtained from the immunoblots. (**E**) Similar to (**A**), except that the WT or *DELE1*$^{-/-}$ MEFs were treated with FCCP (40 μM) for the indicated times.

© The Author(s)

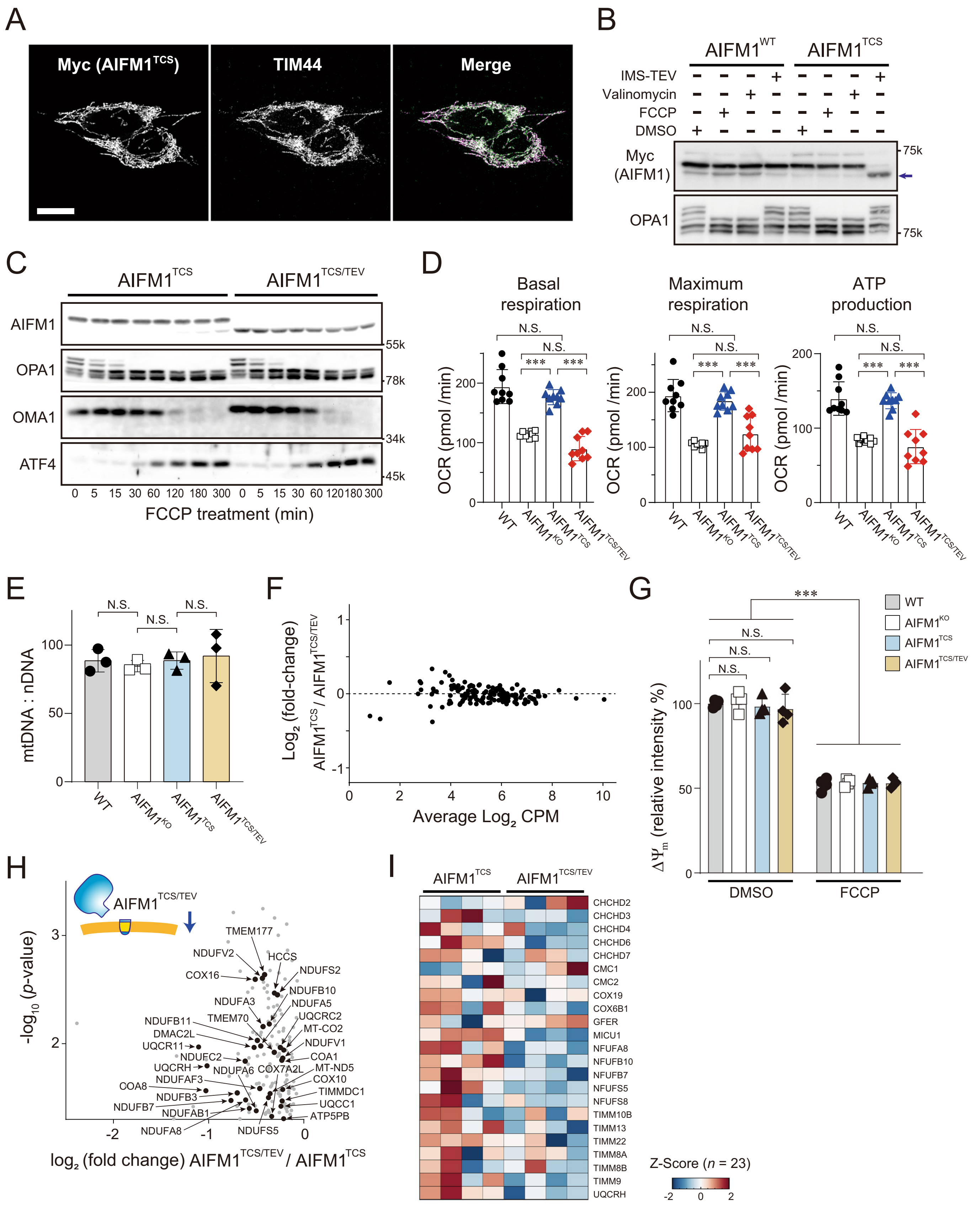

A
Myc (AIFM1TCS)
TIM44
Merge
B
AIFM1WT
AIFM1TCS
IMS-TEV
Valinomycin
FCCP
DMSO
Myc (AIFM1)
OPA1
75k
C
AIFM1TCS
AIFM1TCS/TEV
AIFM1
OPA1
OMA1
ATF4
55k
78k
34k
45k
0 5 15 30 60 120 180 300 0 5 15 30 60 120 180 300
FCCP treatment (min)
D
Basal respiration
Maximum respiration
ATP production
N.S.
***
OCR (pmol /min)
WT
AIFM1KO
AIFM1TCS
AIFM1TCS/TEV
E
mtDNA : nDNA
N.S.
F
Log2 (fold-change) AIFM1TCS / AIFM1TCS/TEV
Average Log2 CPM
G
WT
AIFM1KO
AIFM1TCS
AIFM1TCS/TEV
ΔΨm (relative intensity %)
DMSO
FCCP
N.S.
***
H
AIFM1TCS/TEV
-log10 (p-value)
log2 (fold change) AIFM1TCS/TEV / AIFM1TCS
TMEM177
NDUFV2
HCCS
NDUFS2
COX16
NDUFB10
NDUFA3
NDUFA5
NDUFB11
TMEM70
UQCRC2
DMAC2L
MT-CO2
UQCR11
NDUFV1
NDUFC2
COA1
UQCRH
NDUFA6
COX7A2L
MT-ND5
NDUFAF3
COX10
COA8
TIMMDC1
NDUFB3
UQCC1
NDUFB7
NDUFAB1
ATP5PB
NDUFA8
NDUFS5
I
AIFM1TCS
AIFM1TCS/TEV
CHCHD2
CHCHD3
CHCHD4
CHCHD6
CHCHD7
CMC1
CMC2
COX19
COX6B1
GFER
MICU1
NFUFA8
NFUFB10
NFUFB7
NFUFS5
NFUFS8
TIMM10B
TIMM13
TIMM22
TIMM8A
TIMM8B
TIMM9
UQCRH
Z-Score (n = 23)

Figure EV4. Characterization of AIFM1^{TCS} and AIFM1$^{TCS/TEV}$.

(**A**) Subcellular localization of AIFM1^{TCS}. Flp-In-293-AIFM1^{TCS}/Myc cells were monitored by immunofluorescence against the Myc epitope to determine its subcellular localization (left). Mitochondria in the same cells were also identified by staining with an anti-TIM44 antibody (middle). We confirmed that both AIFM1^{TCS} (magenta) and TIM44 (green) were completely merged in the mitochondria (right). Scale bar, 10 µm. (**B**) The Flp-In-293-AIFM1^{WT}/Myc or -AIFM1^{TCS}/Myc cells were treated for 3 h with either FCCP (40 µM) or valinomycin (1 µg/mL) and analyzed by immunoblotting with the indicated antibodies. AIFM1^{TCS}/Myc cells were resistant to the FCCP/valinomycin treatment but able to convert to the cleaved form in the presence of IMS-TEV (right lane). Arrow, cleaved AIFM1 product. (**C**) AIFM1^{TCS}- or AIFM1$^{TCS/TEV}$-expressing cells incubated with FCCP (40 µM) were collected at the indicated time points (0, 5, 15, 30, 60, 120, 180, and 300 min) and analyzed by western blot. In this experiment, we confirmed that OPA1 processing as well as OMA1 and ATF4 activations were similar in both cell types, indicating that the TEV protease targeted to the IMS had no significant off-target effect. (**D**) Basal respiration, maximal respiration, and ATP production were calculated from the OCR experiment of *AIFM1* KO cells (white), cells expressing AIFM1 variants (AIFM1^{TCS} [blue] and AIFM1$^{TCS/TEV}$ [red]), and Flp-In-293 WT cells (black). Data shown are mean ± SD ($n = 9$ biologic replicates). ***$p < 0.001$ and N.S., not significant (by one-way ANOVA followed by Tukey's multiple comparisons test). The exact p values are summarized in Appendix Table S2. See also Fig. 5H. (**E**) Analysis of mtDNA copy number per nuclear DNA (nDNA) in WT, *AIFM1* KO, and AIFM1 variant Flp-In-293 cells. Data shown are mean ± SD ($n = 3$ biologic replicates). N.S. not significant (by one-way ANOVA followed by Tukey's multiple comparisons test). The exact p values are summarized in Appendix Table S2. (**F**) Representative MA plot of expressed OXPHOS-related genes (MitoCarta3.0) in AIFM1^{TCS}- or AIFM1$^{TCS/TEV}$-expressing cells from transcriptome data. The x-axis represents the $\log_2$ transform of counts per million (CPM), and the y-axis represents the $\log_2$ transform of the fold-change of each gene in AIFM1 variant cells ($n = 1$). A total of 151 of OXPHOS-related genes from the transcriptome data were used in this graph. Expression changes greater than or less than twofold are considered significant, but we identified no genes meeting these criteria. See also Dataset EV5. (**G**) Comparison of the $\Delta\Psi_m$ in WT, *AIFM1* KO, and AIFM1 variant Flp-In-293 cells treated without (DMSO) or with FCCP. Fluorescence values obtained by measuring TMRM were normalized to the average intensity of DMSO-treated WT cells. Data shown are mean ± SD ($n = 3$ biologic replicates). ***$p < 0.001$ and N.S., not significant (by two-way ANOVA followed by Tukey's multiple comparisons test). The exact p values are summarized in Appendix Table S2. (**H**) Enlarged box area in Fig. 6A. A total of 123 mitochondrial proteins (MitoCarta3.0) are plotted and 32 of the OXPHOS subunits decreased in AIFM1$^{TCS/TEV}$ cells are labeled. See also Dataset EV4. (**I**) Alterations in MIA40-pathway in AIFM1^{TCS}- and AIFM1$^{TCS/TEV}$-expressing cells. The mitochondrial proteome was sorted by MIA40 substrates (Reinhardt et al, 2020). Heatmap (Z-scores, $n = 23$): minimum (− 2), blue; maximum (2), red. See also Dataset EV4.

 © The Author(s)

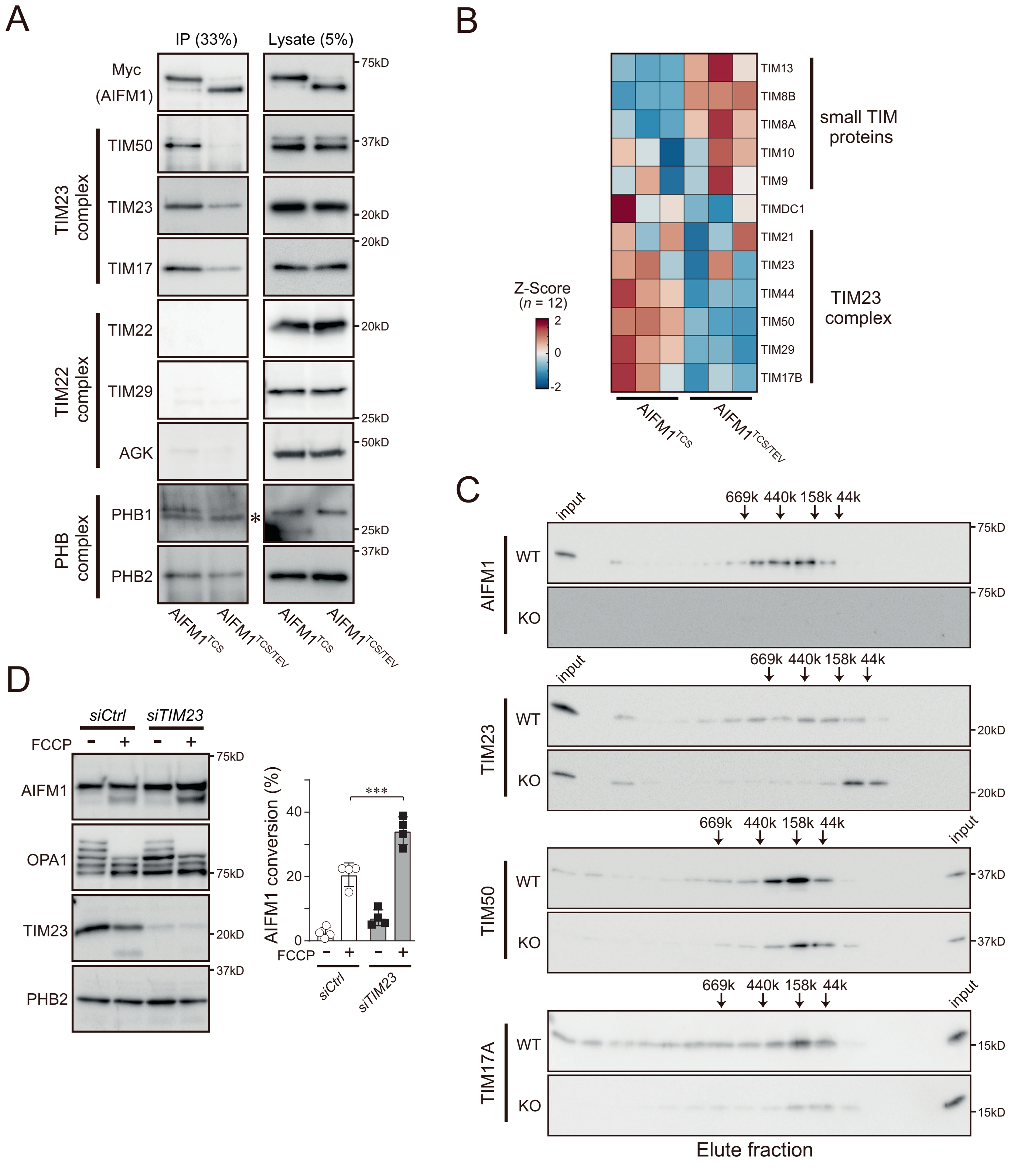
A
IP (33%)
Lysate (5%)
Myc (AIFM1)
TIM23 complex
TIM50
TIM23
TIM17
TIM22 complex
TIM22
TIM29
AGK
PHB complex
PHB1
PHB2
75kD
37kD
20kD
20kD
20kD
25kD
50kD
25kD
37kD
AIFM1TCS
AIFM1TCS/TEV
B
TIM13
TIM8B
TIM8A
TIM10
TIM9
TIMDC1
TIM21
TIM23
TIM44
TIM50
TIM29
TIM17B
small TIM proteins
TIM23 complex
Z-Score (n = 12)
2
0
-2
C
input
669k 440k 158k 44k
AIFM1
WT
KO
TIM23
TIM50
TIM17A
75kD
20kD
37kD
15kD
Elute fraction
D
siCtrl
siTIM23
FCCP
AIFM1
OPA1
TIM23
PHB2
AIFM1 conversion (%)
40
20
0
***

Figure EV5. AIFM1 functions as an assembly factor of the TIM23 translocase.

(**A**) Mitochondrial extracts from the AIFM1 variant (AIFM1^{TCS} and AIFM1$^{TCS/TEV}$) cells were immunoprecipitated with anti-Myc antibody and subjected to immunoblotting using the indicated antibodies. The IP and lysate samples were loaded at 33% and 5% of the input samples, respectively. Asterisk indicates non-specific band. (**B**) Heatmap (Z-scores) showing relative enrichment of selected proteins as identified by IP-MS ($n = 3$) in Fig. 5A. Components of the TIM23 complex were enriched in the AIFM1^{TCS} precipitates, whereas small TIM proteins showed a higher affinity for the membrane-dislocated AIFM1 variant, AIFM1$^{TCS/TEV}$. Heatmap (Z-scores, $n = 12$): minimum (− 2), blue; maximum (2), red. See also Dataset EV3. (**C**) Gel filtration elution profile of endogenous AIFM1 and each subunit of TIM23 complex extracted from the mitochondrial fraction of Flp-In-293 WT cells and *AIFM1* KO cells. The positions corresponding to the elution of standard markers molecular mass and input of samples are indicated, and fractions were analyzed by western blotting with indicated antibodies. (**D**) HeLa cells transfected with siRNA against *TIM23* were treated for 3 h with or without FCCP (40 μM) and analyzed by immunoblotting as indicated. The graph on the right shows the quantification of AIFM1 bands from the immunoblot analyzed by densitometry. Data shown are mean ± SD ($n = 4$ biologic replicates), and ***$p < 0.001$ (by one-way ANOVA followed by Tukey's multiple comparisons test). The exact p values are summarized in Appendix Table S2.

 © The Author(s)

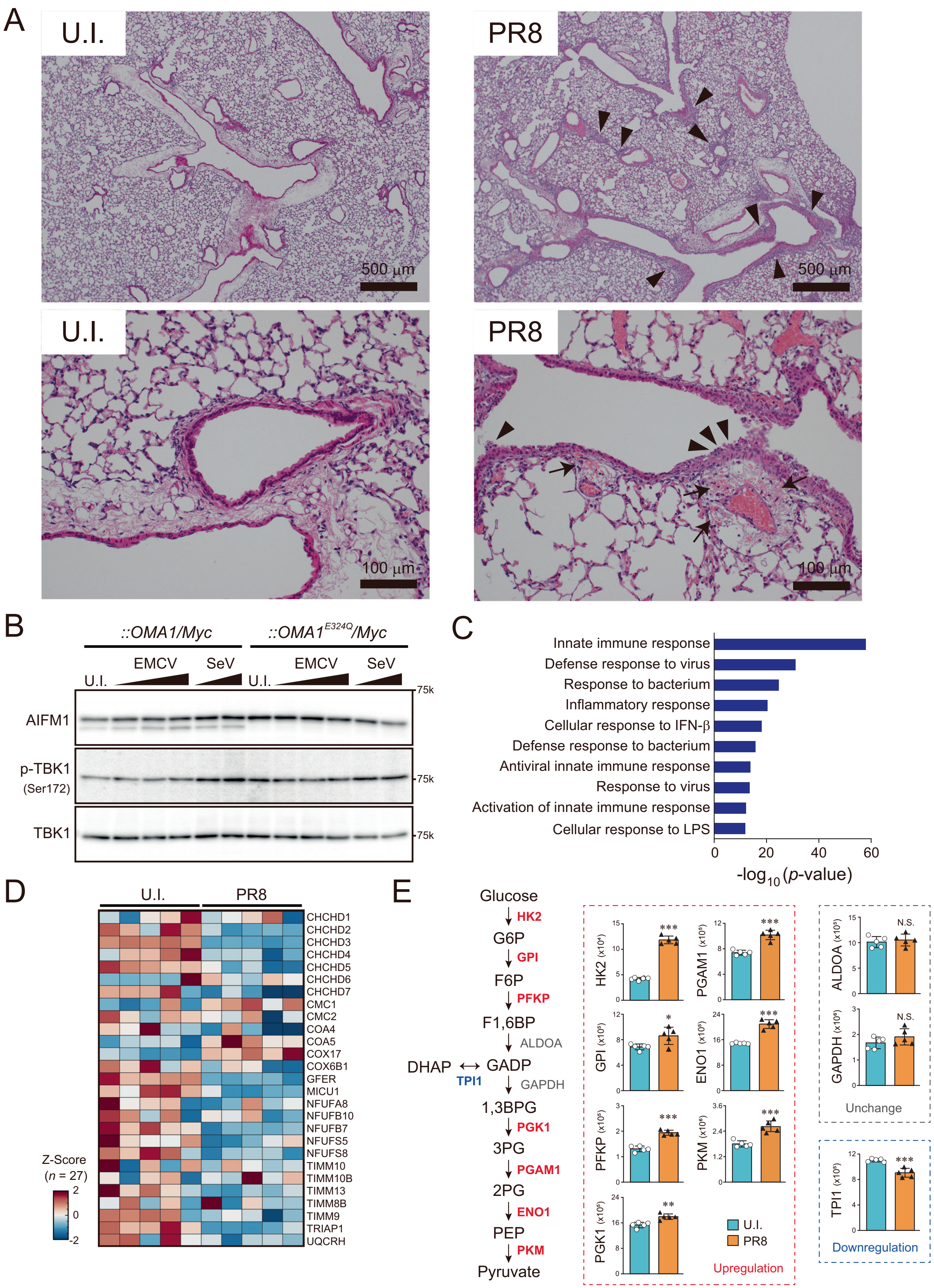

A
U.I.
PR8
500 µm
U.I.
PR8
100 µm
B
::OMA1/Myc
::OMA1E324Q/Myc
EMCV
SeV
U.I.
AIFM1
p-TBK1
(Ser172)
TBK1
75k
C
Innate immune response
Defense response to virus
Response to bacterium
Inflammatory response
Cellular response to IFN-β
Defense response to bacterium
Antiviral innate immune response
Response to virus
Activation of innate immune response
Cellular response to LPS
0 20 40 60
-log10 (p-value)
D
U.I.
PR8
CHCHD1
CHCHD2
CHCHD3
CHCHD4
CHCHD5
CHCHD6
CHCHD7
CMC1
CMC2
COA4
COA5
COX17
COX6B1
GFER
MICU1
NFUFA8
NFUFB10
NFUFB7
NFUFS5
NFUFS8
TIMM10
TIMM10B
TIMM13
TIMM8B
TIMM9
TRIAP1
UQCRH
Z-Score
(n = 27)
2
0
-2
E
Glucose
HK2
G6P
GPI
F6P
PFKP
F1,6BP
ALDOA
DHAP ↔ GADP
TPI1
GAPDH
1,3BPG
PGK1
3PG
PGAM1
2PG
ENO1
PEP
PKM
Pyruvate
HK2 (x10³)
PGAM1 (x10³)
GPI (x10³)
ENO1 (x10³)
PFKP (x10³)
PKM (x10⁵)
PGK1 (x10³)
U.I.
PR8
Upregulation
ALDOA (x10⁵)
GAPDH (x10⁶)
N.S.
Unchange
TPI1 (x10⁵)
Downregulation

Figure EV6. Functional relevance of AIFM1 processing in lungs.

(**A**) On day 7 post-infection (1000 pfu), the lungs were obtained from each PR8-infected or uninfected (U.I.) mouse, sectioned, and analyzed for histopathology following hematoxylin and eosin staining. The lower two panels show higher magnification images. The arrowheads in the PR8-infected lungs indicate detachment of the bronchial epithelium, and the arrows show perivascular hemorrhage. Scale bars, 500 µm (top) and 100 µm (bottom), respectively. (**B**) *OMA1*$^{-/-}$ MEFs that stably express OMA1/Myc or OMA1^{E324Q}/Myc were either infected with SeV (4 or 10 HAU/mL) or EMCV (MOI of 0.1, 0.5, or 1) for 16 h, and the cellular lysates were analyzed by immunoblotting with the indicated antibodies. U.I. uninfected. (**C**) GO enrichment analysis of the whole proteome from the top 10 upregulating biologic processes in PR8-infected lungs (by modified Fisher's exact test). The exact *p* values are summarized in Appendix Table S2. See also Dataset EV6. (**D**) Alterations in MIA40-pathway in uninfected (U.I.) and PR8-infected lungs. The mitochondrial proteome was sorted by MIA40 substrates (Reinhardt et al, 2020). Heatmap (Z-scores, $n = 27$): minimum (-2), blue; maximum (2), red. See also Dataset EV6. (**E**) PR8 infection induces glycolysis in lungs. Quantitative DIA intensities of glycolytic enzymes from the whole proteome analysis were plotted. The left panel shows a schematic overview of glucose metabolism and altered glycolytic enzymes (red, upregulated; blue, downregulated; gray, unchanged). Data shown are mean ± SD ($n = 5$). *$p < 0.05$, **$p < 0.01$, ***$p < 0.001$, and N.S., not significant (by Student's *t* test). The exact *p* values are summarized in Appendix Table S2. See also Dataset EV6.

 © The Author(s)
